# Augmented Reality for Human–Robot Collaboration and Cooperation in Industrial Applications: A Systematic Literature Review

**DOI:** 10.3390/s22072725

**Published:** 2022-04-01

**Authors:** Gabriel de Moura Costa, Marcelo Roberto Petry, António Paulo Moreira

**Affiliations:** 1 Department of Electrical and Computer Engineering, Faculdade de Engenharia da Universidade do Porto (FEUP), 4200-465 Porto, Portugal; amoreira@fe.up.pt; 2 INESC TEC—Institute for Systems and Computer Engineering Technology and Science, 4200-465 Porto, Portugal; marcelo.petry@inesctec.pt

**Keywords:** augmented reality, human–robot collaboration, human–robot cooperation, mixed-reality

## Abstract

With the continuously growing usage of collaborative robots in industry, the need for achieving a seamless human–robot interaction has also increased, considering that it is a key factor towards reaching a more flexible, effective, and efficient production line. As a prominent and prospective tool to support the human operator to understand and interact with robots, Augmented Reality (AR) has been employed in numerous human–robot collaborative and cooperative industrial applications. Therefore, this systematic literature review critically appraises 32 papers’ published between 2016 and 2021 to identify the main employed AR technologies, outline the current state of the art of augmented reality for human–robot collaboration and cooperation, and point out future developments for this research field. Results suggest that this is still an expanding research field, especially with the advent of recent advancements regarding head-mounted displays (HMDs). Moreover, projector-based and HMDs developed approaches are showing promising positive influences over operator-related aspects such as performance, task awareness, and safety feeling, even though HMDs need further maturation in ergonomic aspects. Further research should focus on large-scale assessment of the proposed solutions in industrial environments, involving the solution’s target audience, and on establishing standards and guidelines for developing AR assistance systems.

## 1. Introduction

The personalized production paradigm that characterizes industry 4.0 allows customers to order unique products, with characteristics that the customer himself specifies. The linear, sequential and standardized production lines that emphasize the high productivity and product flow required by the last (third) industrial revolution no longer satisfy the consumer’s current needs and personalized demands. The industry, however, is not yet completely prepared to deal with this paradigm [1,2,3].

While matching this new trend and managing the demand for innovative products, industries are expected to keep high production levels without decreasing their product quality, to maintain relevance within a competitive market. To do so, a high level of production flexibility, decentralization of decision-making, and intelligent use of available resources are required to allow customized mass production without significantly increasing production costs [1,2,4]. Industrial automation alone is no longer enough to fulfill these new requirements. Traditional robotic systems alone are no longer suitable for every task that each production requires. This urge created a path for new technologies, such as collaborative robots and augmented reality, and concepts, such as human–robot collaboration and smart operator or operator 4.0, to emerge.

Even though factories are increasingly employing smarter and connected technologies, human workers still have a dominant role in the production process. Combinations of humans and machines have the potential to outperform either working alone since their capabilities complement each other. For example, humans can handle uncertainties that demand cognitive knowledge and dexterity while robots supply higher physical strength and precision. This duality leads to an idealization of a system where humans and robots combine strengths in a way that both contributors could make use of their values to support each other and not be limited by their conditions, and work towards achieving a common goal. This combination of working is addressed as Human–Robot Collaboration (HRC) [5].

To safely cope with human operators, industrial robots, working in a mechanized repetitive production line, are now being replaced by collaborative robots or cobots. The ISO 10218-2:2011 standard defines collaborative robots as robots designed for direct interaction with a human within a limited collaborative workspace. The same ISO defines a collaborative workspace as a common area within the safeguarded ground where both humans and robots can perform tasks simultaneously during production operations. Hentout et al. [6] presents a comparison between traditional standard industrial robots and industrial collaborative robots, where the main differences between them can be summarized in three major points: the ability to safely interact with humans in a shared workspace, allocation flexibility (light-weight structure, smaller size), and ease of programming.

It is worth mentioning that robots are not collaborative per se. Instead, applications can be rendered collaborative by adopting adequate safety features. Moreover, the term “collaborative robots” suggests the erroneous idea that the robot is intrinsically safe to cope with the operator in all situations [7]. For example, a robot working with a sharp edge as an end effector continues to be dangerous even if working at a low speed and with limited torque.

There are plenty of different collaborative robots manufacturers currently on the market, for example, ABB [8], Kuka [9], Rethink Robotics [10], Universal Robots [11], COMAU [12], Franka Emika [13], Yaskawa [14], TECHMAN [15], among many others. Villani et al. [16] compiled and compared some models specifications in his work. Collaborative Robots can be utilized in numerous industrial applications such as Assembly (screwdriving, part insertion …) [17]; Dispensing (glueing, sealing, painting …) [18]; Finishing (sanding, polishing …) [19]; Machine Tending (CNC, Injection mold …) [20]; Material Handling (Packaging, Palletizing …) [21]; Material removal (grinding, deburring, milling …) [22]; Quality Inspection (Testing, Measuring …) [23]; Welding [24] and many more.

Understanding that the transition from a purely manual or automated production activity to a human–robot cooperative scenario must be done in a way that brings not only benefits to the manufacturing industry (less processing time, increased quality and control of production processes …) but also to the operators (improvement of working conditions, improvement of skills and capabilities …), Romero et al. [25,26], addresses the “smart operator” topic from a human–centered perspective of industry 4.0. The author defines Operator 4.0 as an intelligent and skilled operator who performs work, aided by machines if and as needed. Romero et al. [26] also describes the typologies of operator 4.0 that were compiled, later on, by Zolotová et al. [27], into the definition of:

- Smart operator: an operator that benefits from technology to understand production through context-sensitive information. A smart operator utilizes equipment capable of enriching the real-world with virtual and augmented reality, uses a personal assistant and social networks, analyzes acquired data, wears trackers and works with robots to obtain additional advantages.

According to Milgram and Kishino [28], Augmented Reality (AR) is in between real and virtual reality (VR), thus being considered a mixed reality technology (MR). They define AR as a real environment “augmented” by means of virtual (computer graphic) objects. Azuma [29] defined AR to be any system to have the following properties, which avoid limiting AR to specific technologies, such as head-mounted displays, as previous authors did:

- Combine real and virtual objects;
- To be interactively and in real time;
- To be registered in three dimensions.

Thus, it is possible to rewrite this definition as a technology capable of enhancing a person’s physical environment perception through interactive digital information virtually superimposed on the real world.

Despite not totally mature for every industrial applications due to hardware factors such as ergonomic aspects like weight and movement restraint, which do limit the number of components and features that a visualization equipment can have, due to software factors such as tracking and recognition technology limitations, lack of standards on applications development and on how and which information should be displayed to the user, as well as due to being a new technology yet to be fully integrated into enterprise resource planning systems (ERP) and manufacturing execution systems (MES) [30,31], AR has been used in plenty of different industrial applications such as: Assembly support [32]; Maintenance support [33]; Remote assistance [34]; Logistics [35]; Training [36]; Quality Control [37]; Data visualization/feedback [38]; Welding [39]; Product design/authoring [40]; and Safety [41] among other applications including Human–Robot Collaboration that will be discussed next. Thus, proving that AR is a promising tool to increase the operator’s performance and safety conditions, increase activities execution speed, decrease the error rate during task execution, rework and redundant inspection, and reduce mental workload.

AR visualization technologies can be classified into four main categories as displayed in Table 1 [30,31,42,43,44].

Another important requirement of AR applications is the activation and tracking method, which can be divided into two main categories: marker-based and markerless systems. As the name implies, the main difference between the two technologies is that the first relies on fiducial physical markers, such as QR codes, for example, to be positioned where it wants the virtual information to be anchored and exposed to the user. Although this method tends to be easier to implement, it can suffer in industrial environments due to dirt, corrosion, and poor light conditions. In contrast, markerless systems do not depend on fiducial markers; instead, they rely on objects natural feature tracking, such as borders and colors, which require a much higher processing capacity due to the more computationally intensive, complex, and elaborated detection algorithms, and can also be affected by poor light conditions. For example, one markerless tracking could continuously scan the environment and compare each frame to a CAD model to find a similar pattern or a gesture, whereas a marker-based tracking system scan for a single specific predefined pattern that will trigger the virtual information to be rendered and shown to the user [45,46]. Moreover, tracking systems are important to ensure the accuracy and repeatability of mixed reality systems, which is indispensable for some applications, such as programming by demonstration [47].

With the continuously growing usage of industrial collaborative robots, the urge for achieving a seamless human–robot interaction has also increased. Seamless cooperation between a human operator and a robot agent is a key factor to reaching a more flexible, effective, and efficient production line by reducing the number of errors and time required to finish a task while improving the operator’s ergonomics at work, lowering its cognitive load, enabling decentralized decisions and making better use of available resources.

As a prominent and prospective tool for aiding human operators to understand robots’ intentions and interact with them, Augmented Reality, is expected to fill this gap and solve the “human in the loop” integration problem by augmenting information properly into the operators’ field of view and acquiring its inputs to the cyber-physical system. AR has been demonstrated to be effective for Human–Robot Interaction in numerous fields of applications such as: Assembly guidance [48,49]; Data visualization/feedback [50]; Safety [51]; Programming/authoring [52,53], and so on. The growing market expectations of both technologies [54,55] corroborates with the upward tendency in the number of research papers towards the field of augmented reality human–robot collaboration.

**Table 1:** AR visualization technologies and its characteristics.

Visualization Technology	Examples	Characteristics
Head-Mounted Displays (HMDs)	Comprises smart glasses and helmet devices such as Microsoft HoloLens [56] and Epson MOVERIO [57]	Head-Mounted Displays use mainly optical see-through technology, where the virtual content is superimposed on the operator’s field of view through a transparent display in front of his eyes, allowing the user to see both the real world and the digital information. This technology is also portable and different from Hand-Held Devices, it is hands-free. Its drawbacks consist of bulkiness and eyestrain, being somewhat cumbersome to use for long periods.
Hand-Held Displays (HHDs)	Comprises devices such as tablets and smartphones	Hand Held Displays works by utilizing a video see-through, where the device’s built-in camera records frames of the real world that are then augmented with virtual content. These devices are widely used due to their low cost, easy deployability, usability, and portability, meaning that they are not restricted to stationary applications. However, it has the worst drawback of all, which is to require the operator to hold it during usage. Being a non-hands-free technology makes it not ideal to be applied to manual applications. In addition, it also requires some operator’s attention shift from the task.
Fixed or Static Screens	Comprises devices such as televisions and computer screens	Fixed screens display an augmented real-world view on a stationary monitor. It needs a combination of external image capturing devices and processing tools to generate and display the augmented content. Its drawbacks are the fact that it requires the operator to shift its attention from the task, and it is limited to a stationary application since a large screen is not easily portable.
Spatial Displays	Comprises projection equipment	Projectors display augmented content directly over the real objects and similarly to fixed screens they also need to be combined with external image capturing devices and processing tools. Differently from fixed screens, they do not require the user to shift its attention from the task though it is also limited to a stationary application. Both modalities facilitate collaborative tasks among users since they are not associated with a single user, differently from the following technologies. One possible drawback of this modality is that, when using a single projector, it can suffer from occlusion problems. It is also known as Spatial Augmented reality (SAR).

Previous reviews compiled the current state of the art of Augmented Reality for industrial applications, and enlighten the advancements and current challenges faced towards its implementation in production systems. Danielsson et al. [58] raised the current maturity level of Augmented Reality Smart Glasses (ARSG) for industrial assembly operations and highlighted the endeavors required before large-scale implementations, either from a technological and engineering perspective. Makhataeva and Varol [59] focused on compiling several AR applications, from 2015 to 2019, for medical applications, motion planning and control, human–robot interaction, and multi-agent systems, providing an overview of AR, VR, and MR research trends in each category. Hentout et al. [6] presented a literature review of works on human–robot interaction in industrial collaborative robots between 2008 and 2017. Bottani and Vignali [60] reviewed the scientific literature concerning AR applied to the manufacturing industry, from 2006 to early 2017, where the authors organized 174 papers according to publication year, publication country, keywords, application field, industrial sectors, technological devices, tracking technology, user test, and results. De Pace et al. [4], brought together papers about augmented reality interfaces for collaborative industrial robots, from 2001 to 2019, and analyzed them to find the main usage and typologies of AR for collaborative industrial robots, the most analyzed objective and subjective data for evaluating applications, as well as the most adopted questionnaires for obtaining subjective data, also to identify the main strengths and weaknesses of AR usage for these applications, and finally to point out future developments regarding AR interfaces for collaborative industrial robots.

This systematic literature review, differently from those previously mentioned ones, intends to address AR applications specifically applied to human–robot cooperative and collaborative work. For a paper to be considered, it must include an application where both humans and robots work together either in the same task or in different ones simultaneously while sharing a common workspace.

The purpose of this review is to categorize the recent literature on Augmented Reality for Human–Robot Collaboration, published from early 2016 to late 2021, to examine the evolution of this research field, identify the main areas and sectors where it is currently deployed, describe the adopted technological solutions, as well as highlight the key benefits that can be achieved with this technology.

The remainder of this article is organized as follows. Section 2 presents some terms definitions adopted in this work. Section 3 describes the research methodology adopted for the literature survey and presents some preliminary information about the studies analyzed. Section 4 details the survey results and includes not only descriptive statistics on the sample of papers reviewed but also their categorization and their detailed analysis. Finally, Section 5 summarizes the key findings from the review, discusses the related scientific and practical implications, and indicates potential future research. In the end, it also depicts and outlines this work’s final considerations.

## 2. Definitions

According to Vicentini [7], self-explaining terms like collaboration, cooperation, and collaborative robots can be prone to misguided and inconsistent use in industrial technical communication. Within this context, this section presents the definitions used throughout this paper.

**Figure 1:**
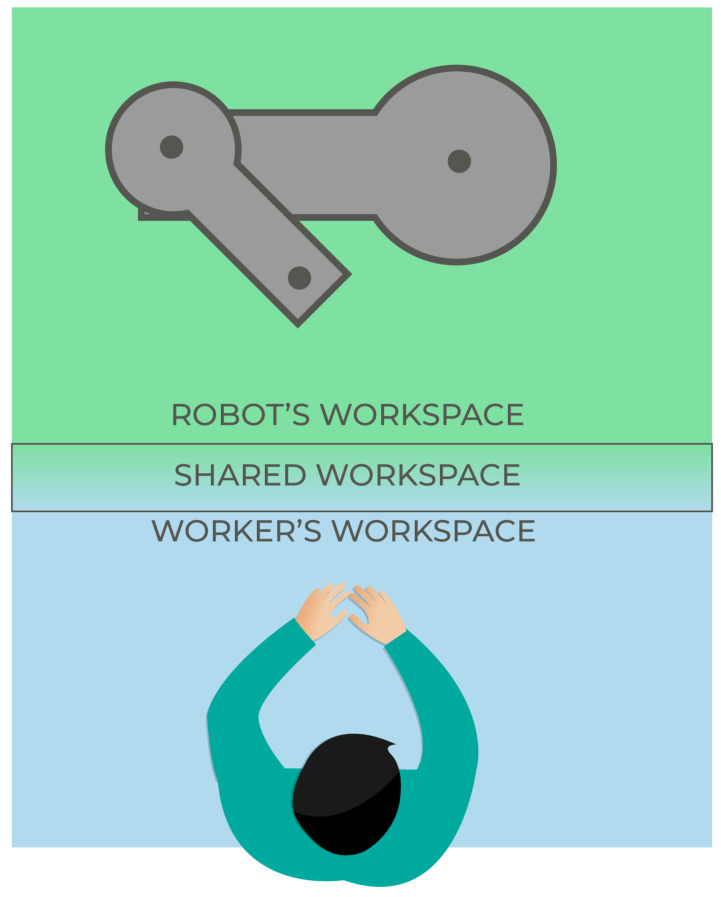
Workers’ and robots’ workspaces.

**Figure 2:**
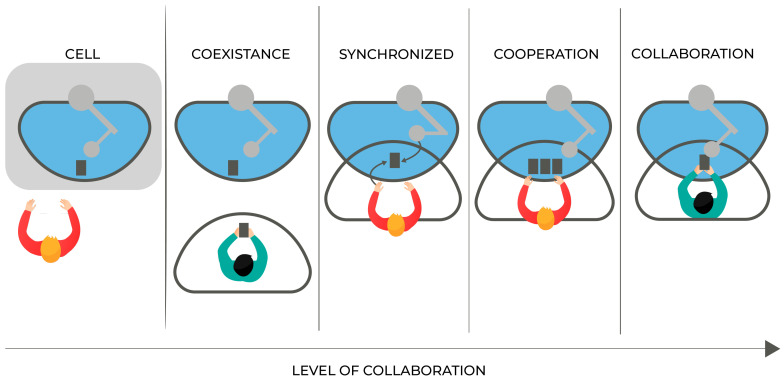
Levels of Interaction.

- Collaborative robot/cobot: a robot designed for direct interaction with a human within a defined collaborative workspace [61].
- Collaborative/shared workspace: a space within the safeguarded space where the robot and a human can perform tasks simultaneously during production operation [61]. In other words, the overlapping space in between the human and the robot’s reach (Schmidtler et al. [62] apud. Thiemermann [63]), Figure 1.
- Human–Robot Interaction: general term for all forms of interaction between humans and robots (Schmidtler et al. [62] apud. Bortot [64]). Can be classified in five levels of interaction (cell, coexistence, synchronized, cooperation, collaboration), Figure 2.
- Cell: Not a genuine cooperation scenario since the human and the robot are separated by a safety cage [65].
- Coexistence: when the human operator and the cobot are in the same environment but have separated workspaces [66].
- Synchronised: when the human operator and the cobot work in the same shared workspace, but at different times [66].
- Cooperation: when the human operator and the cobot work in the same shared workspace at the same time, though each element of this interaction performs a different task [66].
- Collaboration: when the human operator and the cobot work in the same shared workspace, executing the same shared task at the same time [66].

**Figure 3:**
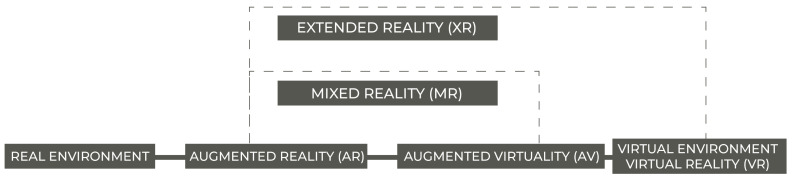
Extended Reality continuum.

As can be seen in Speicher et al. [67], unlike the concept of Virtual Reality (VR), the concepts of Augmented Reality (AR) and Mixed Reality (MR) are still blurry when it comes to a standardized definition. Therefore, the following definitions, adapted from Milgram and Kishino [28], Azuma [29], Chang et al. [68], Drascic and Milgram [69], Fast-Berglund et al. [70], Maas and Hughes [71] and Alizadehsalehi et al. [72], will be used throughout this paper:

- Virtual reality (VR): Immerses users in a completely artificial digital environment Maas and Hughes [71].
- Augmented reality (AR): overlays virtual objects on the real-world environment and also provides the ability to interact with that environment Azuma [29]. According to Milgram and Kishino [28], AR is any case in which an otherwise real environment is “augmented” by means of virtual (computer graphic) objects.
- Augmented Virtuality (AV): Enhance virtual reality with real-world feedback [69].
- Mixed reality (MR): A broader term that encompasses either Augmented reality and Augmented Virtuality and is used as a general term to refer to a blending of the virtual and the physical world [68,70].
- Extended Reality (XR): An even broader term for referring to all real-and-virtual combined environment and human–machine interactions generated by computer technology and wearables. In other words, it is a collective term for immersive technologies, encompassing all reality definitions presented before [70,72]. The extended reality continuum is depicted in Figure 3.

## 3. Methodology

To assess the current use of Augmented Reality in industrial human–robot collaborative and cooperative operations, a Systematic Literature Review (SLR) approach has been adopted. Fink [73] defined SLR as a “*systematic, explicit, and reproducible method for identifying, evaluating, and synthesizing the existing body of completed and recorded work produced by researchers, scholars, and practitioners*”. It helps to collect all related publications and documents that fit pre-defined inclusion criteria to answer a specific research question. It uses unambiguous and systematic procedures to minimize the occurrence of bias during searching, appraisal, synthesis, and analysis of studies [74]. This previous course of action is also known as the SALSA framework. Based on De Pace et al. [4] rearrangement of Grant and Booth [75] SALSA Framework, the procedure presented in Table 2 was adopted to guide the execution of this systematic review. This framework ensures the replicability and transparency of this study [76]. For this research, Parsifal [77], an online tool to support systematic literature reviews that provides mechanisms to help design and conduct, was used.

### 3.1. Protocol

The Population, Intervention, Comparison, Outcome, and Context (PICOC) framework is a useful strategy to determine the research scope, which helps to formulate research questions, determine the research boundaries, and to identify the proper research method [4,74]. Other similar works have also applied this methodology, such as Fernández del Amo et al. [78] and Khamaisi et al. [79]. This paper PICOC framework is presented in Table 3.

The resulting objectives are presented in the form of research questions in the list below:

1. What are the main AR visualization technologies used in industrial Human–Robot collaboration and cooperation context?
2. What is the main field of application of AR in industrial Human–Robot collaboration and cooperation context?
3. What is the current state of the art of AR applications for Human–Robot collaboration and cooperation? Is research focusing on experimental or concept applications? What are the most used assessment techniques and indicators? What are the research gaps presented in AR for industrial Human–Robot collaboration and cooperation context?

Having defined the questions to be answered by this SLR, the following subsections will describe the adopted procedure used throughout the conducting of this research.

### 3.2. Search

The search phase consists of identifying sources of information that could be relevant for this research and defining the search string. Even though some search engines, such as Scopus and Web of Science, encompass overlapping publication venues [80], it was decided to use multiple different databases to assert their relevance when searching this specific field of study, by comparing how many of the selected papers can be found in each one. Therefore, the selected search engines were: ACM Digital Library [81], Dimensions [82], IEEE Xplore [83], Web of Science [84], ScienceDirect [85], Scopus [86] and Ebsco [87].

During the search phase, it was brought to attention the aforementioned plurality of interpretations of some relevant terms to the research. Thus, based on the combination of main keywords (Augmented Reality—Mixed Reality—Cooperation—Collaboration), multiple strings were created to try to fill this gap and cover the largest number of papers, preventing any relevant article from being left out. Those strings are generally presented below, and some minor modifications were needed for specifics search engines that do not support wildcards and to adequate the search for Title, Abstract and Key-words fields. They are:

- ((“Augmented reality” OR “AR”) AND (“human–machine collaborati*” OR “HMC” OR “human–robot collaborati*” OR “HRC”))
- ((“Mixed reality” OR “MR”) AND (“human–machine collaborati*” OR “human–robot collaborati*”))
- ((“Augmented reality” OR “AR”) AND (“human–machine cooperati*” OR “human–robot cooperati*”))
- ((“Mixed reality” OR “MR”) AND (“human–machine cooperati*” OR “human–robot cooperati*”))

It is relevant to point out that abbreviations were only used for the first search string. After the first search, it was realized that those abbreviations were not reaching relevant articles; instead, they were reaching a large number of papers out of this research scope. Therefore, they were discarded for the following search strings. The search was realized on 3 January 2022.

### 3.3. Appraisal

The appraisal step comprises the filtering and quality assessment of the search results. The main goal of a quality assessment procedure is to identify a paper selection criterion in case two or more studies present similar approaches or conflicting ideas or results [4]. The study selection consisted of the screening of search results for selecting papers that were relevant to this review. To make the selection process repeatable, a set of pre-determined exclusion criteria were defined, and are presented in Table 4.

Based on similar reviews, such as De Pace et al. [4], and using the proposed search strings on the Scopus database, it is possible to infer that the augmented reality for Human–Robot Collaboration and Cooperation topic, despite having its first papers published by 2004, started gained relevance by 2016, where the number of publications consistently surpassed the threshold of two papers per year. Therefore, the year 2016 was chosen as the starting year of this review.

Nowadays, search engines offer several filters capable of narrowing, at source, the search scope. Year and research field filters were applied to every database, according to its respective criteria, described in Table 4, resulting in a reduced number of 378 articles. After screening and applying all exclusion criteria to these 378 papers found, the selected (remaining) papers had their references manually searched and compared against the exclusion criteria in a second search phase. This second search phase returned nine potential papers that added up to the 387 papers presented in Table 5.

A two-step selection process was applied for selecting the relevant papers. A first sieve was made among the articles found, where the title, the abstract, the results, and the conclusions were analyzed, which resulted in a total of 42 papers. Then, a second and more thorough and in-depth read of the 42 remaining papers was carried out to confirm whether these papers did not present evidence of fitting the exclusion criteria in other sections that were not previously analyzed. After this second step, a total of 32 papers remained.

Afterward, a quality assessment procedure has been applied to evaluate the selected papers. According to Fernández del Amo et al. [78], in case any biases or contradictory ideas appear when analyzing selected-relevant papers, quality assessment results can be used to provide more transparent, repeatable findings. Eleven Quality Criteria (QC), presented in Table 6, were chosen for the quality assessment procedure. QC1 and QC2 are quantitative metrics extracted from the databases and represent the normalized number of citations per year and the normalized impact factor, respectively.

The Normalized number of citations per year (QC1) is defined by the equation:

$$\begin{array}{cc} \mathrm{QC}1 & =\frac{C_{p}}{MaxC_{y}} \end{array}$$

where $C_{p}$ is the paper number of citations and $MaxC_{y}$ is the largest number of citations among the selected papers within the same publication year. This was used as a metric to compare the relevance of each paper in comparison to others published in the same year.

In addition, the Normalized Impact Factor (QC2) is defined by the equation:

$$\begin{array}{cc} \mathrm{QC}2 & =\frac{SJR+JCR+CS}{Max_{sum}} \end{array}$$

where SJR is the impact factor from Scimago Journal Rank, JCR is the impact factor from Clarivate Analytics Journal Citation Reports, CiteScore (*CS*) is the impact factor from Elsevier, and $Max_{sum}$ is the highest number obtained from the sum of those three impact factors. This was used as a measure to compare the relevance of each journal.

QC3 to QC10, also presented in Table 6, are subjective metrics and their score are derived from the authors’ analysis of the selected papers. All the 32 selected papers were evaluated against each criterion with a score of 0 (no compliance), 0.5 (partial compliance), or 1 (full compliance).

Table 7 presents the main author country and the paper publication year. The selected paper’s quality assessment is presented in Table 8, detailing the score attributed to each paper, either to the quantitative and qualitative criteria. It is important to note that these results were not used further in this review since no bias or contradictions were found in the analysis of the 32 papers. Despite that, according to Fernández del Amo et al. [78], the quality assessment is a process prone to bias itself; therefore, the mean and standard deviation for each criterion is provided as a tool for numerical validation of a potential author bias in the assessment process. Not considering the quantitative criteria (QC1 and QC2), the papers that were perceived with the subjectively higher quality were Kalpagam et al. [49] and Chan et al. [88], followed by Materna et al. [89], Hietanen et al. [51], and Tsamis et al. [90], with a score of 7.5 out of 8 for the first two, and 7 out of 8 for the following three, respectively. If one considers the quantitative criteria, the order of relevance changes to Hietanen et al. [51], Kalpagam et al. [49], Chan et al. [88], Materna et al. [89], and Michalos et al. [91] figuring out in the last place instead of Hietanen et al. [51] on the top 5, for a small score difference of 0.025 points. This last place change may or may not revert, due to the quantitative criteria, as well as other papers’ classifications, over time, since newer papers will eventually be more cited in future works.

In Table 9, the papers that do not have a database associated with it as “Found by the search string” were found when searching the references of selected papers and added manually; therefore, they were not found by the search string. Moreover, special attention must be paid to the Terminology columns, where the “String Combination” column represents the combination of keywords used on the search string to find the paper, and the third column represents the classification of the papers, whether it is a cooperative or collaborative application according to the definitions presented in Section 2. Since the terminology of mixed reality used in this work encompasses the augmented reality definition, there is no point in classifying the papers according to reality enhancement terminology.

### 3.4. Syntheses and Analysis

The synthesis and analysis phase consisted of a thorough examination of the selected papers for extraction and classification of data that could further be organized to highlight relevant information to the research scope. To do so, several topics were identified to be analyzed; they are: Application field, Robot type (collaborative or not-collaborative) and User interaction mechanisms; Visualization and Tracking methods; Research type and Evaluation techniques; Demographic (Authors, Country, and publication year); and, finally, Database, Publication venues, and Associated Terminology.

The evaluation of these synthesized data is expected to draw out discussions and conclusions that can answer the proposed research questions. It also aims to map how each theme was covered by each paper, find a correlation between them and identify the research field poles, evolution over time, and search mechanisms.

Table 10 presents some of this information extracted from the selected papers. Please note that Lee et al. [97], in the Robot column, does not mention what robot was used, and where there is only the manufacturer, as in papers Andersen et al. [93], Liu and Wang [94], Mueller et al. [102], Bolano et al. [106], Lotsaris et al. [111], and Andronas et al. [113], the author does not specify what robot was used, but it was possible to identify the robot manufacturer through figures.

## 4. Systematic Literature Review Analysis

### 4.1. Database, Publication Venues and Terminology

As can be seen in Table 9, several papers have been found in multiple databases, indicating that there are some overlaps. Out of the 32 selected papers, 26 were found by the research strings. All of those 26 papers could be found on the Scopus search engine. Web of Science was the database with the second most found papers, followed by IEEE Xplore, as can also be seen from Table 9.

**Figure 4:**
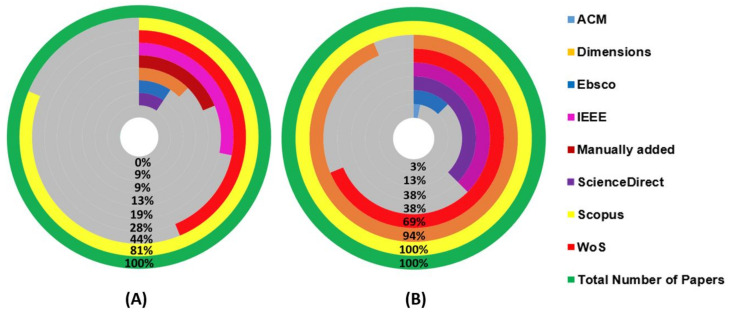
(**A**) Number of articles found by the search string in each database; (**B**) number of selected articles that could have been found in each database (search string + manual search).

**Figure 5:**
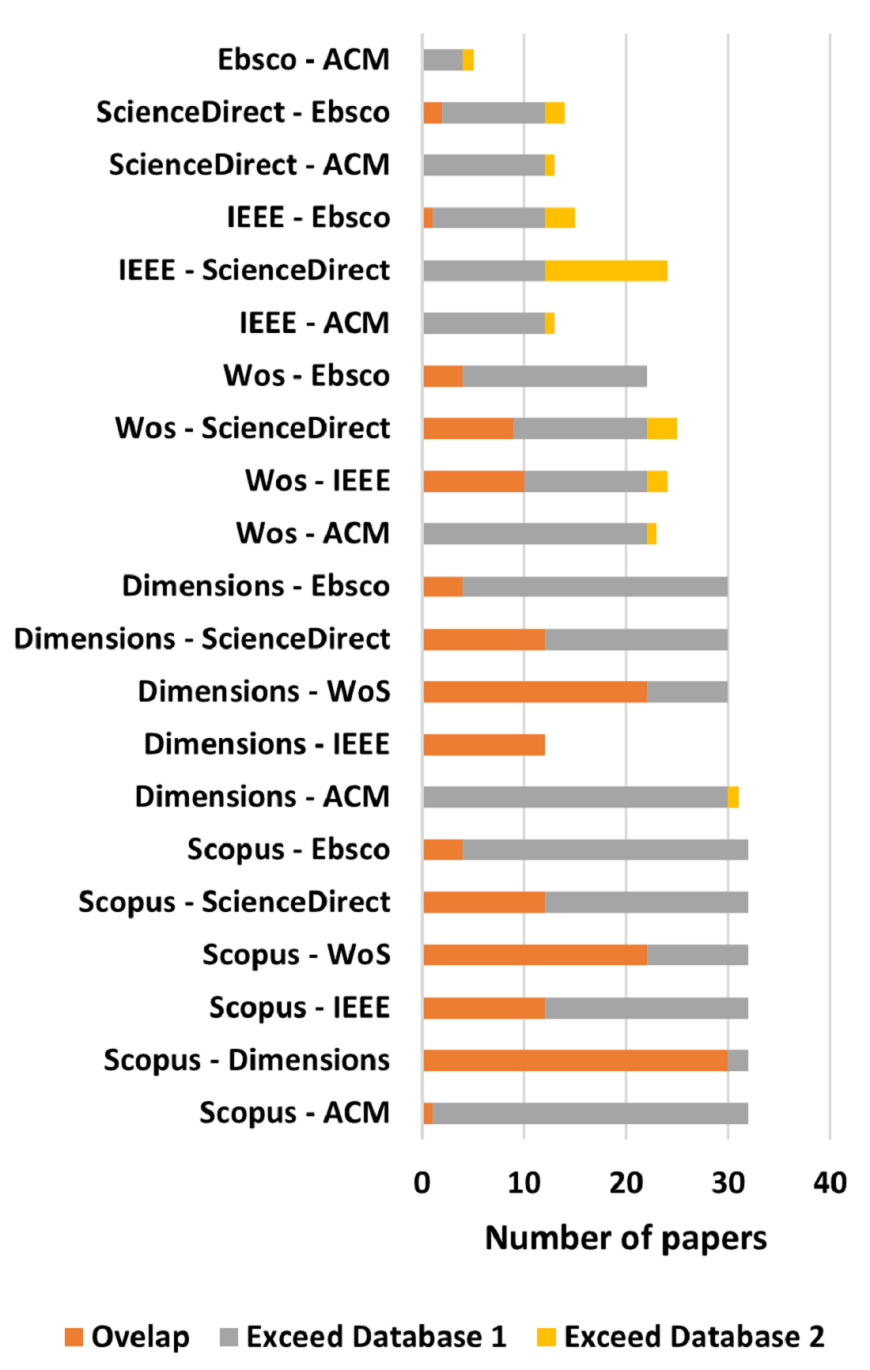
Database overlap.

Using the title of each selected paper a manual search was done in every database to verify if the databases contained a selected paper that was not found by the search string. Figure 4A shows the percentage relative to the number of papers found by the search string in each database while Figure 4B shows the number of selected papers that could have been found in each database. Figure 5 shows the overlap between them, from which it is possible to infer that using just one of the databases does not guarantee that every paper regarding a subject will be covered.

One of the reasons why the databases did not find all selected papers might be because some of those that were manually added did not have the search keywords on the searched fields (title, keywords and abstract) due to author’s choice, probably because the searched terms were not the focus of the work. Moreover, one was under the Human–Robot Interaction keyword, which if searched would have returned a numerous amount of papers out of the scope of this research.

From these figures, it is possible to conclude that, for this specific field of study, there is no need for searching relevant articles in multiple databases, since most of them can be found using the Scopus search engine. Although other databases, such as Dimensions.ai, also have a great number of papers in their database, the search engine somehow could not find as many papers as Scopus did, using the same search string. There can be two reasons for this: either the search string is too restrictive, or the Dimensions’ search engine is not as flexible as Scopus or Web of Science ones.

Understanding that Dimensions, Scopus, and Web of Science are indexing services databases, it was expected that they would find most of the papers, which did not happen with the Dimensions one when using the search string, as mentioned before. Regarding the other databases, which provide archival services, the one that presented the most papers when searched with the proposed search strings and with the manual search was the IEEE Xplore.

**Figure 6:**
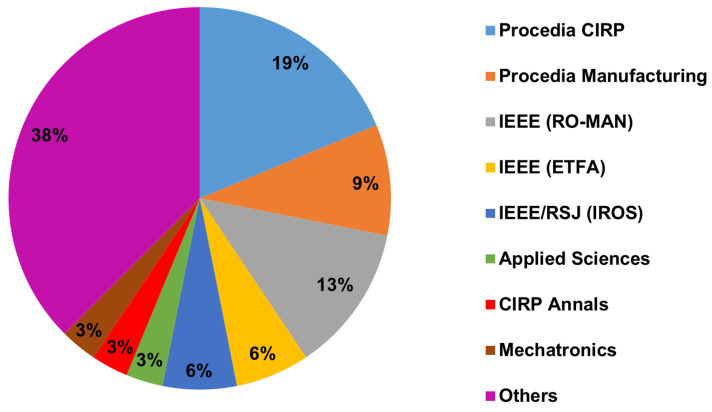
Publication venues.

With respect to publication venues, the one with most papers found was Procedia CIRP, followed by IEEE International Conference on Robot & Human Interactive Communication (RO-MAN) and Procedia Manufacturing, respectively. Details can be seen in Table 7 and in Figure 6.

**Figure 7:**
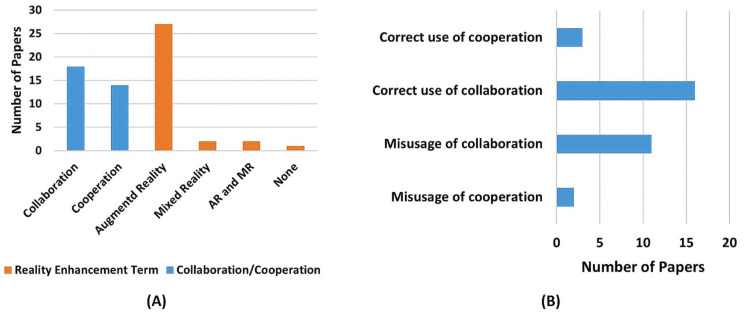
(**A**) number of papers by term; (**B**) correct usage of collaboration and cooperation according to Section 2 terminology.

The string combination, second column, in Table 9 presents the combination of terms that were used on the search string for finding that paper. Section 2 definition, third column, presents the classification of the papers application as cooperative or collaborative according to the definitions presented in Section 2. As can be noticed in Figure 7A, the most commonly used terms for each category are Augmented Reality and Collaboration.

It also shows that, despite blurry to standard definition [67], the usage of the term AR is more widespread for referring to reality enhancement technology than the term MR. On the other hand, Figure 7B corroborates Vicentini [7] the statement that the terms’ cooperation and collaboration are prone to misguided usage. Figure 7B was achieved by comparing the selected papers used, cooperation or collaboration, and terms to describe the activity, against the definitions presented in Section 2.

In the end, 18 papers were classified as collaboration and 14 as cooperation, by evaluating their application according to the definitions presented in Section 2. This can be seen in Table 9.

### 4.2. Demographic

For extracting bibliometric networks, the VOSviewer [116] software tool was used. These networks may for instance include publication venues, researchers, or individual publications, and they can be constructed based on citation, bibliographic coupling, co-citation, or co-authorship relations. Based on co-occurrence Analysis, where the relatedness of items is determined based on the number of documents in which they occur together, and on citation analysis, where the relatedness of items is determined based on the number of times they cite each other, some interesting conclusions can be drawn.

**Figure 8:**
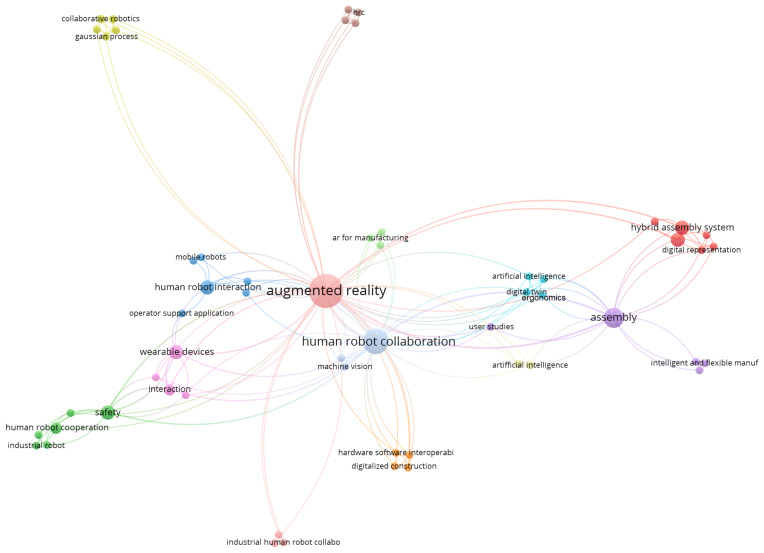
Author keywords’ co-occurrence correlation.

From Figure 8, which describe the co-occurrence correlation between the authors used keywords, it is possible to infer that the Augmented Reality and Human–Robot Collaboration terms that have the largest number of co-occurrence, as expected; on the other hand, the terms Human–Robot Cooperation and Mixed Reality did not have as great an influence in comparison with the first two. Another conclusion that can be dragged out from this figure is that Assembly and Safety applications concentrate the greatest amount of research on AR for the human–robot collaboration and cooperation field.

**Figure 9:**
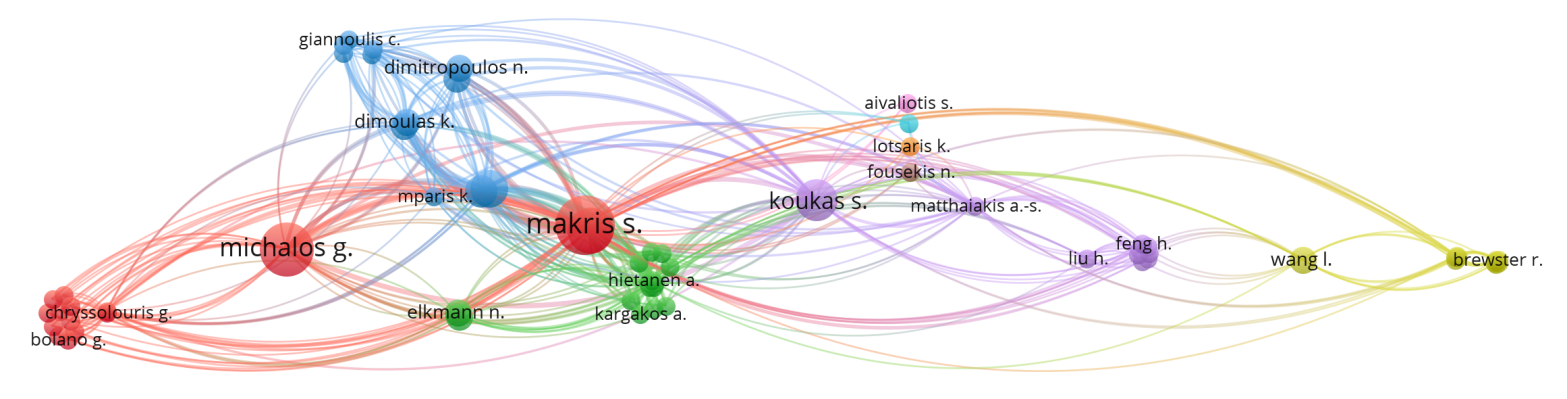
Author citation correlation between the selected papers.

**Figure 10:**
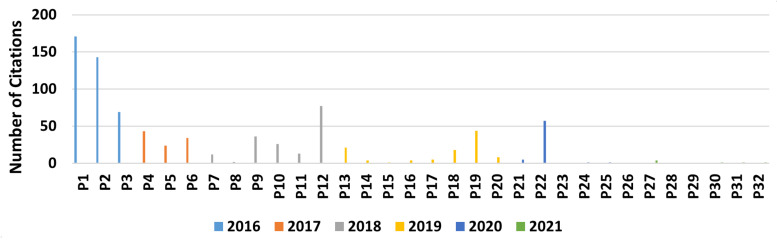
Paper citations by publication year.

**Figure 11:**
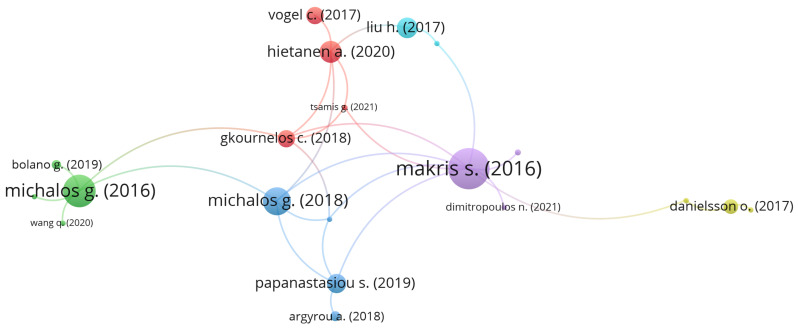
Document citation correlation between the selected papers.

**Figure 12:**
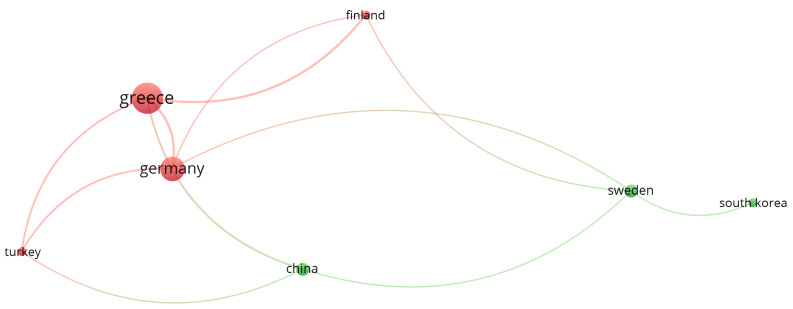
Country citation correlation between the selected papers.

Regarding Figure 9, which presents the author citation correlation between the selected papers, it is possible to identify that Makris, Michalos, and Karagianis are the most referenced authors, among the selected papers. This is because the three of them are co-authors on very cited papers, such as Makris et al. [92] (171 citations) and Michalos et al. [91] (143 citations), as can be seen in Figure 10 and the authors’ correlation in Figure 11. This influence can also be seen in Figure 12, where Greece is the most cited country followed by Germany. Another fact that corroborates with this last statement is the number of papers found by country, which can be seen in Table 11, where Greece and Germany have the largest number of selected papers followed by the United States and China. If related works were counted as one, Greece’s number of papers would be reduced to 6, shortening the distance between the countries but not changing the current ranking. In the future, when a larger sample might be available for analysis, the country with the most published papers might alternate according to the willingness of researchers of each country to further study the augmented reality for human–robot collaboration and cooperation topics.

**Figure 13:**
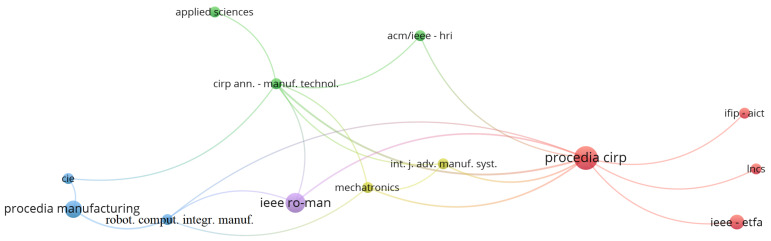
Publication venues’ citation correlation.

With respect to publication venues, the most cited one was Procedia Cirp, followed by IEEE ROMAN and Procedia Manufacturing, which could be expected since they are the top three publication venues with the most found papers and also have a significantly high impact factor, as can be seen in Figure 13.

**Figure 14:**
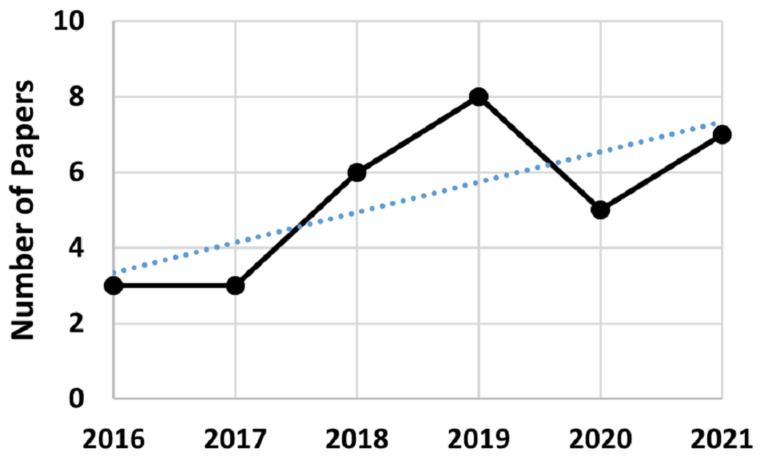
Papers per year. The blue line shows the linear trend line regarding the number of publications per year on AR for HRC context.

Finally, Figure 14 presents the number of papers per year. It is possible to infer from its trend line that, in this moment, it is not a fast growing research field, slowly attracting the interest of researchers and yet to be considered matured.

### 4.3. Robot Type

**Figure 15:**
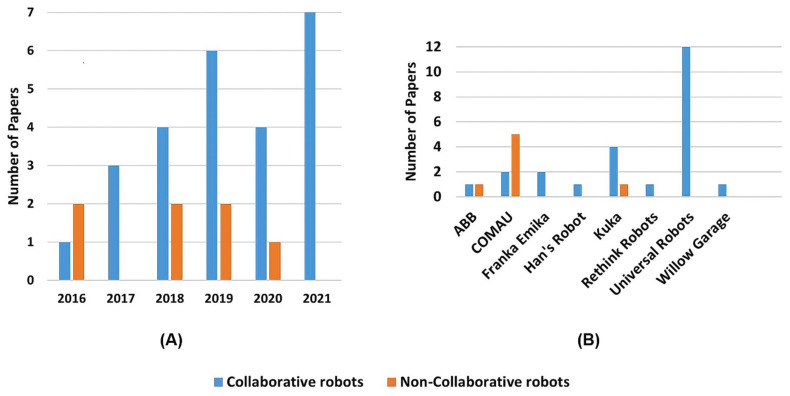
(**A**) Type of robots per year; (**B**) papers by robot manufacturer.

Figure 15A clearly shows that the research focus, regarding the augmented reality for human–robot collaboration and cooperation field, is growing towards the usage of collaborative robots, while there is not as much interest in cooperative usage of non-collaborative robots. Regarding the selected papers, 78% use collaborative robots while only 22% developed works using non-collaborative ones.

Even though non-collaborative robots can be used for cooperative applications, if correctly configured with features to provide a safe interaction, Figure 15A may sustain researchers’ interest in maintaining industrial non-collaborative robots to mechanized repetitive burdening tasks, where there is no need for human intervention. In addition, the fact that most non-collaborative high payloads robots are large eventually makes them cumbersome for collaborative tasks in smaller workspaces, being used only for larger applications only.

Figure 15B shows a clear researcher’s preference towards Universal Robots cobots usage for collaborative applications. This may be because UR is one of the main manufacturers of collaborative robots and the existence of open-sourced application programming interfaces (APIs) for control and simulation of robot actions, as well as establishing remote or local communication with the robot. As for non-industrial robots, COMAU is the leading manufacturer used in research for human–robots’ collaboration and cooperation.

#### 4.3.1. Non-Collaborative Robots

Makris et al. [92], Gkournelos et al. [98] and Michalos et al. [99] use a non-collaborative COMAU NJ 130 robot as a means of aiding the operator to load a car axle and rear wheel group to fixtures, where the human operator can perform the necessary tasks. Similarly, Michalos et al. [91] uses a COMAU NJ 370 for the same application of handling a rear-wheel group to fixtures. Gkournelos et al. [98] also uses a COMAU Racer 7 non-collaborative robot for applying sealant in specific parts of a refrigerator, in an assembly operation, and so does Papanastasiou et al. [105]. All of the above-mentioned papers are related to each other, thus being extensions of the same work.

On the other side, Ji et al. [101] uses an ABB IRB 1200 non-collaborative robot for assembling smaller machined parts in the same assembly station, while Vogel et al. [109] uses a Kuka KR60L45 non-collaborative robot for aiding the operator on transferring workpieces from one station to another.

#### 4.3.2. Collaborative Robots

Andersen et al. [93] used a UR cobot for a pick and place task, where the robot and the operator, alternately, change the position of a box over a table. On the other hand, Tsamis et al. [90] also uses a UR manipulator for a pick-and-place task, but for exchanging parts from a workstation to a different one. Liu and Wang [94], Argyrou et al. [96], Hietanen et al. [51] uses a cobot to aid the operator on a car engine assembly process, performing parts handling and mounting. Similarly, Danielsson et al. [48] on its turn uses a UR3 for assisting the operator on a wooden car toy assembly task. In the same model, Kalpagam et al. [49] uses a UR5 robot to support the operator on a car door assembly task. Mueller et al. [102] uses a UR cobot for an aircraft riveting process, where the robot would correctly position the anvil while the operator handled the riveting hammer. Bolano et al. [106] uses it to screw bolts on a metallic part, while the operator places the bolts on the next part. Wang et al. [107] uses a cobot to aid the operator on an automotive gearbox assembly. Lotsaris et al. [111] uses cobots to aid in the process of assembling the front suspensions of a passenger vehicle, by moving and lifting heavy parts. Finally, Andronas et al. [113] uses a UR manipulator to assist the operator to assemble a powertrain component, performing the lifting of heavy parts and fastening operations.

Vogel et al. [95], Paletta et al. [103], Kyjanek et al. [104] and Chan et al. [88] made use of Kuka collaborative robots. Vogel et al. [95] uses a Kuka iiwa LBR 14 for assisting the operator to screw mounting plates to a ground plate in a cooperative assembly task, while Paletta et al. [103] uses a Kuka iiwa LBR 7 on a toy problem, where the cobot assists the operator on a pick and place task, where the goal is to assemble a tangram puzzle (toy problem). Kyjanek et al. [104] employs a Kuka cobot to support the human operator on a non-standard wooden construction system, where the cobot position and holds the part in the correct location while the operator attaches them. Finally, Chan et al. [88] works with a Kuka manipulator on a large-scale manufacturing task, where the robot and the human operator work together on an aircraft pleating task.

Lamon et al. [100] and De Franco et al. [50] employed Franka Emika’s Panda cobot for two different applications. Both authors use the cobot for assisting the operator in the assembly of aluminium profiles, by holding the piece in the right location while the operator attaches them. Moreover, De Franco et al. [50] also presents a second application where the robot and the human operator work collaboratively on a surface polishing task, where the operator guides the manipulator, with a polishing tool as the end effector, controlling the amount of force applied over the surface. These papers are related to each other and thus can be considered an extension of each other regarding the aluminium profile assembly operation.

Dimitropoulos et al. [114] and Dimitropoulos et al. [115] utilize two different COMAU collaborative robots. Both Dimitropoulos et al. [114] and Dimitropoulos et al. [115] use the high payload collaborative robot COMAU AURA for assisting the human operator on mounting an elevator, more specifically, the cab door panel hangers, where the robot lifts the heavy parts and position them in an ergonomic position for the operator to perform the necessary tasks. Dimitropoulos et al. [115] also uses a low payload collaborative robot COMAU Racer 5 to aid the operator in assembling large aluminium panels; in this case, the robot does not perform any lifting; instead, it performs riveting actions around the panel. These papers are related to each other, thus, they can be considered an extension of each other regarding the elevator assembly operation.

For assisting the operator on a stool assembly process, where the robot picks the next part to be assembled from a storage bin and places it over the mounting table, Materna et al. [89] makes use of a Willow Garage’s PR2 robot. Hald et al. [108] for a drawing application (toy problem), where the objective was to assess the operators’ trust towards the robot as the robotic manipulator speed changes, takes on the Rethink Robotics’ Sawyer cobot. Finally, Luipers and Richert [112] uses an ABB YuMi for handover tasks.

### 4.4. Application Field

Although one may argue that assembly operations is a term that comprises smaller activities such as riveting, gluing, even material and tools handling, or even part placement (pick-and-place activity), this paper decided to consider the combination of these smaller activities as one. The same is true to the fact that the pick-and-place activities were considered in this paper to embrace exclusively pick-and-place or handling-only activities.

Therefore, a great dominance of research covering assembly applications over pick-and-place and handover tasks can be noticed with a quick analysis of Table 12, which could be expected since the majority of applications that involves human operators and cobots working together in industrial environments are towards optimizing the production line, where the robot takes care of repetitive and non-ergonomic tasks, while the operator focuses on more dexterous and cognitive activities. Of the 27 assembly applications presented, 74% employed collaborative robots, while 22% used non-collaborative robots. Regarding the five pick-and-place activities presented, four (80%) employed collaborative robots, while only one (20%) implemented a non-collaborative robot. The activities, considered in the “others” column, refers to De Franco et al. [50] polishing and Hald et al. [108] drawing activities. Both made use of collaborative robots. It is important to mention that some papers presented more than one application, therefore being considered once for each application.

**Figure 16:**
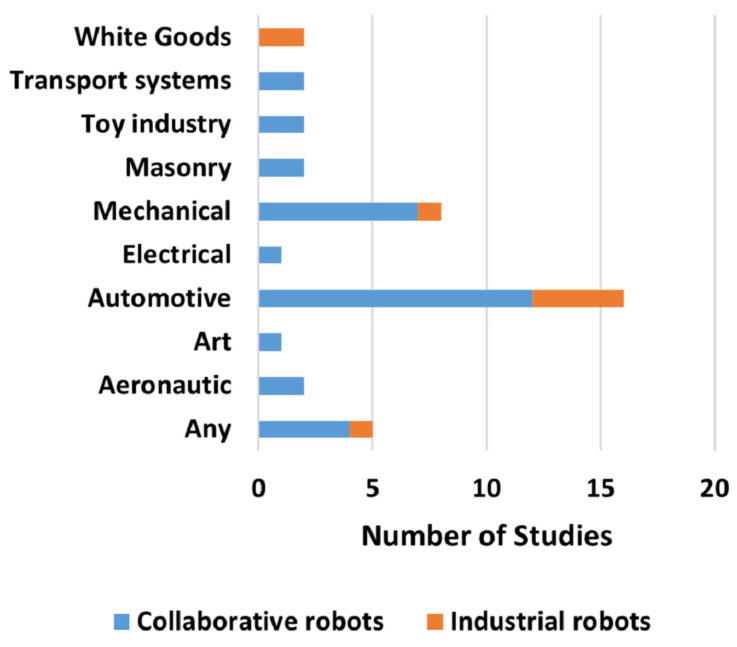
Number of studies by industry.

Moreover, it is possible to notice a greater research interest in industrial automotive applications, which is presented by Figure 16. This may be because the automotive industry has always been a pioneer when it comes to testing emerging technologies and especially to the fact that it presents numerous challenges that are a perfect fit for evaluating different applications, from a small scale to a larger one. The term “any” in Figure 16 indicates that the developed application does not target a specific industry.

**Figure 17:**
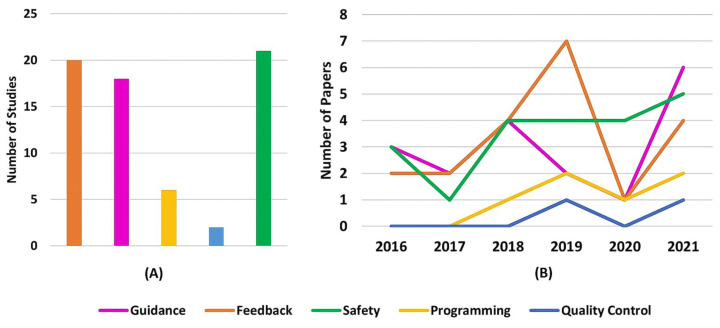
(**A**) Number of studies by applications; (**B**) application fields over time.

Regarding AR applications, it is possible to see three major areas of interest, which can be seen in Table 13 and Figure 17A. It is possible to see researchers’ great interest in the safety research topic, where researchers intend to use Augmented reality for improving, among other factors, the operator’s awareness. Following behind, in second place, is using augmented reality for inserting the human operator in the loop through relevant production and process-related data feedback. Moreover, in the third position, guidance applications highlight the interest in using AR as a tool to support operators throughout tasks.

Furthermore, since Augmented Reality for Human–Robot Collaboration is a growing research field, it is reasonable to expect that all categories have growing tendencies, despite some being steeper than others. This is ratified by Figure 17B.

It is interesting to point out in Figure 17B that the feedback category presents in 2020 a steeper declination contradicting the general upward tendency shown. This counter tendency movement happens since the feedback category was thought of as the application capability to provide extra information that would not fit any other presented categories. Therefore, after analyzing each 2020 selected paper’s features and augmented information, most were classified as Guidance, Safety Programming, or Quality Control. Despite that, in 2021, the feedback category followed the upward trend again. Therefore, it can be considered a sampling noise to the graph.

**Figure 18:**
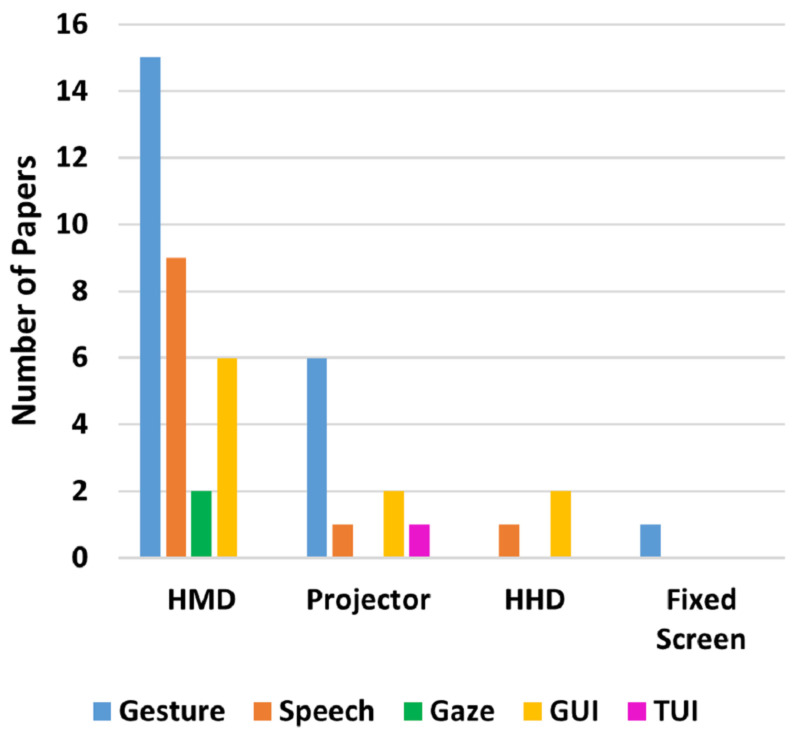
User interaction modalities by visualization method.

Andersen et al. [93], Gkournelos et al. [98], and Dimitropoulos et al. [114] present more than one application and industry; therefore, they were considered multiple times in Figure 17B and Figure 18, one for each different application and industry.

#### 4.4.1. Applications—Safety

This subsection describes the usage of Augmented Reality for enhancing operators’ safety in human–robot collaboration tasks. Aiming to enhance operator’s safety, Makris et al. [92], Michalos et al. [91], Gkournelos et al. [98], Michalos et al. [99], and Papanastasiou et al. [105] use AR to project visual alerts, show the robot’s end-effector trajectory and enable workspace visualization. Those visual alerts can be a warning that the robot has started or stopped moving, a production line emergency stop, or any other general alert for example. The workspace visualization is composed of two holographic cubical safety volumes, one green, indicating the area where it is safe for the human operator to act within, and a red one, indicating the area where the robot will act upon and possible collisions may occur in case the operator enters it. These visual cues increase the operator’s awareness and may help avoid accidents on the shop floor. It is important to mention that those above-mentioned functionalities are for informational purposes only, meaning that the AR system does not detect whether the operator breaches the non-safe space or not, thus not triggering any protective stop or robot speed reduction. For these applications, an external, COMAU C5G controller, double encoder certified system is used to ensure operator safety during operations. In addition, Gkournelos et al. [98] and Michalos et al. [99] use for the automotive application the PILZ Safety Eye [117] 3D camera system. Moreover, Gkournelos et al. [98] and Papanastasiou et al. [105] also use, for the white goods application, the AIRSKIN [118] safety sensors.

Kalpagam et al. [49] enhances operators’ safety perception by projecting onto the workpiece the robot’s intention through warning signs and highlighting the segment on which the robot will act upon, increasing the operator awareness of the robot intentions. Kalpagam et al. [49] also projects warning signs and safety zones on the ground, highlighting in red the robot workspace, in yellow a caution zone, and in green, a safe zone, where there is no risk of immediate harm to the operator, as Vogel et al. [109] also does. The difference between these last two is that, unlike Kalpagam et al. [49], Vogel et al. [109] projects a dynamically changing safety zone and also covers a larger area since it uses a non-collaborative large robot in its application. Another important difference is that Vogel et al. [109] is capable of identifying if the caution/warning zone or the Robot/danger zone is breached, using KEYENCE Safety Laser Scanners [119], and halting the robotic manipulator motion if so.

Using a projector-camera safety detection system, Vogel et al. [95] projects dynamic robot working volumes onto the collaborative workspace. The projector highlights the area that will be occupied by the robot with a striped outline, and when the human operator breaches the safety borders, the camera detects a deviation of the projected lines, triggering a robot motion stop and changing the outline to a solid red color. This increases the operator’s awareness of the robot’s working area and may avoid potential hazards during the operation. Argyrou et al. [96] projects warning signs at the operator’s field of view and on a graphical user interface on the operator’s smartwatch and also triggering safety protocols, based on the input of the application developed HRC monitoring system.

Combining a depth camera-based safety monitoring system with augmented reality, Ji et al. [101] sends as feedback to the operator his distance to the robot and respective warning messages in different colors according to the safety level, and also sets the robot speed accordingly, even bringing the robot to an eventual full stop. In addition to that, visual safety alerts and the robot end-effector trajectory can also be shown to the operator. This application also allows the operator to edit the robot’s trajectory and to preview the motion by visualizing the robot’s digital twin performing the planned trajectory, enabling the operator to set a safer path for the robot avoiding possible collisions. Kyjanek et al. [104] also projects the robot’s end-effector trajectory for the user and allows the user to make a trajectory planning. On the other hand, Chan et al. [88] does not show the robot’s path after planning but keeps the waypoints, set by the operator, visible in a way that the user can have minimal knowledge of the robot working area and displays a digital twin robot so the user can preview the robot motion prior the execution.

To enhance the operator awareness and safety during operation, Bolano et al. [106] projects, on the working table, a green outline around the piece which the robot is currently working on, as well as the specific screw it will tighten. Moreover, it can also render in 2D the robot’s planned trajectory swept volume or the end effector’s planned trajectory, according to the users’ preference. Therefore, the operator can be aware of possible collision areas. In addition to that, the developed system can also detect whether the operator or another obstacle obstructs the robot’s planned motion. It casts a red marker on the detected spot and an additional warning text is displayed to the user explaining the task’s current state, and the robot is stopped.

For monitoring, if the operator breaches the dynamically changing safety zones during the robot operation or not, Hietanen et al. [51] developed a safety monitoring system based on a depth camera. Two approaches were adopted as visualization means of the safety zones. The first approach uses a projector, to render the boundaries of the safety zone in blue over the workstation. The second approach uses AR glasses, through which the safety zones’ boundaries are rendered as a solid virtual fence in red with a pre-determined height. In case of any boundary violation, the robot is led to a full stop. The paper also uses a virtual dead man’s switch for ensuring the operator’s safety when allowing the robot to perform the next actions.

Hald et al. [108] projects over the paper the lines that the robot is going to draw. Similarly, Luipers and Richert [112] also intends to show the user the cobot planned path so the user can be aware of the robot’s intended motion. Schmitt et al. [110] projects in the workstation colored areas, being green and red for human and robot workspaces, respectively, and yellow for a collaborative task, where they share the same working area.

To make the user aware of potential danger, Lotsaris et al. [111] communicates to the user when the manipulator platform starts a movement by displaying augmented notifications. In addition, the operator can visualize planar safety fields around the mobile platform. If any danger zones violation is detected, by the laser scanners, the robot is immediately set to a complete stop. Moreover, the user can see, through the HMD, the digital twin of the mobile platform, and the robotic arms at the trajectory’s final position, as well as a line that indicates the path to be followed.

Tsamis et al. [90] also has a robotic manipulator mounted over a mobile platform. The developed augmented reality application enables the user to visualize the mobile platform and the manipulator’s planned trajectories by interpolating holographic 3D green spheres accordingly to the calculated waypoints. The manipulator’s maximum workspace is also shown to the user as a red semi-transparent red sphere, with its center always on the cobot base. Through virtual collision detection, the application is capable of identifying whether the operator is inside or outside a virtual geometric shape. Thus, this feature is used for detecting if the operator breaches the robot’s workspace. If so, the robot motion is halted and a potential collision warning message is shown to the user. Similar to Chan et al. [88], the application also renders a digital twin of the robot with which the operator can preview the planned motion, but, unlike Chan et al. [88], the user cannot edit the planned path.

Last but not least, Andronas et al. [113] uses augmented reality to show the user the robot trajectories and safety zones. However, differently from other applications, this one uses, in addition to visual warning messages, audio notifications as well.

Some other papers mention safety features but do not integrate them into augmented reality. Liu and Wang [94] uses a depth sensor for monitoring the distance between the robot and the operator, but no AR cues are displayed to the user. Paletta et al. [103] tracks the user head and hands with a motion capturing system and computes the distance to the nearest robot part; based on that and on the participant’s stress level, the robot arm’s speed is re-adjusted downwards to a potential full stop. In addition, Dimitropoulos et al. [114] uses the vibration feature of a smartwatch as cues for safety alerts.

It is important to mention that, even though some approaches are capable of detecting through AR whether the operator breaches a non-safe working area and acts upon the robot by reducing its speed or even stopping it, most of the above-mentioned AR safety functionalities are not certified but still can be used as an extra layer of protection.

Moreover, an interesting classification can be done regarding whether the presented approaches are active or passive agents ensuring the user’s safety. Approaches that are not capable of monitoring the current state of the operation, by meaning of identifying a breach in the safety zones or anticipating a possible collision by monitoring or predicting the operators’ movement and comparing it against the robot current planned trajectory, and thus do not act to prevent an accident by stopping or reducing the robot speed or even changing the robot trajectory on the fly, can be considered passive approaches, while those that are capable of acting upon the system to prevent accidents can be considered active approaches.

Most of the papers, such as Makris et al. [92], Michalos et al. [91], Gkournelos et al. [98], Michalos et al. [99], Papanastasiou et al. [105], Andersen et al. [93], Kalpagam et al. [49], Kyjanek et al. [104], Chan et al. [88], Hald et al. [108], Luipers and Richert [112], Schmitt et al. [110], and Andronas et al. [113], present only safety informational content and do not act directly to prevent accidents or collisions. Therefore, they can be classified as passive approaches. On the other hand, as discussed above, Vogel et al. [109], Vogel et al. [95], Argyrou et al. [96], Ji et al. [101], Bolano et al. [106], Lotsaris et al. [111] and Tsamis et al. [90] presented active approaches towards assuring the operator’s safety.

#### 4.4.2. Applications—Guidance

This section describes the usage of Augmented Reality as a guidance tool to assist the operator in a human–robot collaborative task.Makris et al. [92], Michalos et al. [91], Gkournelos et al. [98], Michalos et al. [99] and Papanastasiou et al. [105] used Augmented Reality to display textual and 3D modeled holographic instructions to the user. Through this feature, the operator can see all needed parts, tools and components for that corresponding task. The 3D holographic model of the components allows the operator to know exactly where and how each part should fit or be used. Gkournelos et al. [98] does not use an AR guidance system for the sealing application.

For instructing the user on how to interact with a tool or the assembly item itself, Andersen et al. [93] developed a system capable of projecting this information directly over the workpiece. It shows whether the operator should rotate it, to which direction and how much rotation was still needed—or whether the operator should move it, highlighting the final destination on the table and also showing the distance left for the manipulation to be successful. The values shown are updated in real time according to the operator’s action over the item.

Using a large screen, Liu and Wang [94] displays assembly text instructions to the operator. Moreover, the developed system uses feature recognition to identify the parts and tools available in the workstation, and, if one matches the stored CAD file, a 3D label is rendered over it to facilitate its recognition by the user. According to the identified item, the correct instructions are also projected to the operator. The application renders some other visual cues such as a red arrow over the next piece to be manipulated by the cobot.

Making usage of augmented text instructions and 3D holographic representation of to be assembled items for highlighting locations and parts of interest in each step Danielsson et al. [48] aims to assist the operator through an assembly process. The instructions are presented in the upper right corner so as not to cover the main part of the screen. Moreover, the highlighted parts or locations could blink or be static.

To aid the operator, Argyrou et al. [96] uses augmented reality to present textual information on a virtual slate and colored arrows to highlight areas of interest and walk the operator through an engine assembly process. The application also uses the developed monitoring system to calculate the status of the current task and display the appropriate instructions.

Kalpagam et al. [49] projects, on the workpiece and the ground, instructions to aid the operator through a door assembly process. This application shows for example how much the operator needs to move or rotate and how much is left for this interaction to be concluded. The values shown were updated in real-time according to the operator’s action over the item. It also shows the operator where to place specific parts and which ones to join. Moreover, it highlights needed tools and locations where the operator should move to during the task. The application also uses a projection mapping detection algorithm to identify when a task was correctly completed by projecting a confirmation sign to the user and automatically passing it to the next step.

Retrieving from a database, Ji et al. [101] makes available to the user stored production instructions by rendering a combination of 3D colored arrows and text pointing at the location/item where the operator/robot should act over. The text has the same color as its respective arrow, and the instructions describe the action, the agent, and the step order.

By projecting information on the table when using the projector approach or through a virtual slate when using the Augmented Reality head-mounted display, Hietanen et al. [51] communicates to the user through images and detailed text instructions describing the task being performed and the robot’s following actions—similarly, Andronas et al. [113], through virtual slate projects’ task information and detailed instructions to the operator. Moreover, it also presents to the user a 3D holographic visualization of the part being assembled, highlighting where it should be mounted. The same concept is presented in Luipers and Richert [112] where the author intends to use an AR feature to guide the user throughout an assembly operation by projecting a textual description of the current step. Lotsaris et al. [111] on its turn makes use of multiple workbenches, so the developed application presents, in addition to the textual description of the task to be performed, indications showing in which workbench the task should be performed.

For the first application, Dimitropoulos et al. [114] projects on top of the aluminum panel being worked with, textual instructions and task description along with illustrations of the parts and procedures that the operator has to execute. In addition, arrows and highlighted shapes indicate where each component should be assembled. The second application uses an HMD device to display to the user, animated 3D models and auxiliary panels containing information about the procedure being executed. It also indicates the position where each part should be assembled, as well as the required tools necessary for executing that task. Using the HMD approach, the user can switch between two levels of assistance, the higher one with detailed information and 3D holographic animations, or choose the basic one that just renders the part contour where it should be assembled. When being used at the highest level of assistance, the system is capable of identifying whether the operator is close to the hologram, disabling the graphics superimposed over the real part to enhance the visibility of the operator. Furthermore, the controller developed by Dimitropoulos et al. [114] is also capable of detecting the operator’s actions and sending notifications if the operator is manipulating the wrong objects or performing wrong actions. In case the operator performs correct actions, the system will also identify it and move to the next step.

Lastly, Dimitropoulos et al. [115] mentions the usage of AR instructions but does not describe how it was shown to the user. In the work, the HMD device is also used as an image capturing system that attempts to identify the step that the operator intends to execute and properly show instructions, as a means to make the user interaction more seamless.

#### 4.4.3. Applications—Feedback

This section describes the usage of Augmented Reality for feedbacking useful information, apart from the previously mentioned guidance and safety data, to the operator in a human–robot collaborative task. Makris et al. [92], Michalos et al. [91], Gkournelos et al. [98] and Michalos et al. [99] provide, upon user request, shop floor status and production data. The received feedback contains information about the current and the next product to be assembled, the average remaining cycle time to complete the current operation, and the number of completed operations versus the targeted one.

In its turn, the system developed in Danielsson et al. [48] shows the user the overall progress during the assembly process, informing the current and the total number of steps. It also shows the operator which voice commands are available in each step. The command text is highlighted in green if it was successfully recognized by the system or in red if the system was not sure about the speech and whether the command has been called or not, and therefore not triggering any function.

Making use of three projected auxiliary areas, one in the bottom plus one on each side of the workspace, Vogel et al. [95] approach, projects textual messages and notifications to the user in case of errors and fault diagnosis and textual instructions on how to handle them. It also shows the user the current state of the robot and the task progress status.

The AR application developed in Lee et al. [97] is capable of providing to the user real-time process data of a specific product, such as assembly blueprint, as well as its current production status. This blueprint is a general process overview, not step-by-step guidance throughout the operation. This is why it was classified as feedback and not as guidance.

On a more human–centered approach, the AR application in Paletta et al. [103] projects into the user’s field of view, his current mental load, and concentration level, through a virtual yellow and orange bar, respectively. It also shows the users’ gaze, represented by a small blue sphere. Similarly, Andronas et al. [113], besides the information presented as guidance or safety features, also gives feedback on the operator’s ergonomics by coloring a virtual small manikin’s body parts with a green, yellow and red color scale. The ergonomic analysis is done individually for each part of the body.

During the execution of the task in Materna et al. [89], the user has access to the current state of the system and error messages, as well as the currently available actions in a notification bar close to the edge of the table. In addition, for error messages, a sound was played as audio feedback to the user. Moreover, for every part detected and picked by the robot, an outline and ID are displayed, where this part will be placed.

With one of the simplest user interfaces among all selected papers, Lamon et al. [100] and De Franco et al. [50] show to the operator just a text and a small sphere. Through the color change of the sphere and display of different texts, the interface is capable of indicating to the user the activity that is being performed by the robot, whether it has grasped an item or not, or even if it is in an idle state, waiting for user input. Furthermore, De Franco et al. [50] presents a similar interface for the polishing task. In this last one, the text was changed for numbers that feedback to the user the magnitude of the force being applied to the surface, and the small sphere was changed for an arrow, which increases or decreases its size according to the amount of force being applied (the higher the arrow, the higher the force). The arrow also follows a green, yellow and red color code that represents how far the applied force is from the desired amount. Both Lamon et al. [100] and De Franco et al. [50] present a beep sound to alert the operator that the robot is moving.

Luipers and Richert [112] presents a concept of an AR feature to give feedback to the user on the assembly status, which steps were concluded or not, as well as the overall progress of the process. Ji et al. [101] goes a little further exhibiting production-related information, such as the running time of the task, the expected remaining time to conclude it, which task is being performed, the current and total number of steps, and how many components are available to be assembled.

Understanding that a lack of coordination between the agents of an aircraft riveting operation parts can severely damage the aircraft fuselage, the Augmented Reality application in Mueller et al. [102] enables the user to see the current robot’s position on the other side of the aircraft fuselage during the riveting process. With a related idea, allowing the user to visualize the robot motion during an automobile gearbox collaborative assembly simulation, Wang et al. [107] enables the user to train, learn the assembly process, and get used to working with a cobot.

By giving the user access to general production-related information, such as the current assembly step, the task completed percentage, the piece ID, how many parts have already been placed, and how many layers have already been built, Kyjanek et al. [104] makes use of the augmented reality HMD communication capability to obtain these pieces of information through ROS messages. Moreover, the user can visualize the robot joints velocity, the exact location where the robot will place the part that is being manipulated, and also the position where the remaining parts should be assembled.

In Bolano et al. [106], items that are within the workspace are outlined according to their current status. If the item is ready and available to be worked with, it is outlined in purple. If the robot is currently working on an item, this item is outlined in green. Lastly, if a possible collision is detected, a red outline is projected. As another feedback feature, the application also projects a representation of the workspace on the left side of the work table, so the operator can have a quick overview of it even if the robot is occupying and occluding the workspace. It also casts on the table textual information about the current state of the robot and error descriptions. This feedback is important to make the operator aware of the current status of the vision system. In this way, the operator can detect instantly if the robot has issues in the localization of the parts placed into the workspace.

In cases of failure, emergency, or exception handling, in Lotsaris et al. [111], the operator gets informed with popup notifications that describe the type of emergency and give specific information on how to overcome it. Moreover, it also provides the user notifications about the activity’s current status. Similarly, in Tsamis et al. [90], the robot’s current status is given as feedback to the user informing whether the motion execution or planning has failed or succeeded.

#### 4.4.4. Applications—Programming

This section describes the usage of Augmented Reality for programming or re-planning the robot trajectory during a human–robot collaborative task.Materna et al. [89] makes use of a touch-enabled table in combination with a projection system to enable the user to program the robot to execute an action more abstractly. It proposes the usage of pre-constructed instruction blocks which can be connected based on the success or failure result of the previous command, allowing for simple branching and cycling of the program. Those blocks were given a simple straightforward name (e.g., “pick from feeder”), and their parameters should be set individually. The programs with set parameters were colored in green, the ones that still had parameters to be set in red, and the ones that do not have parameters in gray. The robot parameters are set by kinesthetic teaching and the final position of the items by dragging the respective outline over the table. The author also simulates, using the Wizard of Oz approach, that the application had a user monitoring system when using two specific instructions (“wait for the user to finish”/“wait for user”). These instructions were expected to make the robot wait for the user to finish executing some activity, or to return to the center of the table, respectively. When executing the program, all instructions become gray and non-interactable.

The robot’s end-effector planned trajectory, in Ji et al. [101], is represented by evenly spaced waypoints represented by small white spheres. The user can edit the initially planned path by making a pinch hand gesture, and dragging this waypoint to the desired location. Every time the user does it, the path is updated to satisfy the user’s modification. By using voice commands, the user can preview the planned trajectory being executed by the robot’s digital twin and, if satisfied, the user can confirm it with another voice command.

Abstracting the user from planning trajectories, Kyjanek et al. [104] uses the ROS-based planning framework MoveIt! [120] for generating the robot’s trajectory. In this way, by using a pre-programmed robot’s behaviors and spatial limitations in combination with the known position and shape of the already assembled structure, pressing the plan button triggers a generation and preview of a collision-free and valid robot motion. After the user’s confirmation, the planned path is sent to the robot controller for execution as a set of joint values.

To create a trajectory for the robot, in Chan et al. [88], the user wearing the HMD looks at a point on the work surface and uses the voice command “set point” to set a waypoint. A virtual sphere is rendered at the desired position with an arrow indicating the surface normal. At any moment, the user can say the “reset path” command to clear any set waypoints. After done planning, the operator can use the “lock path” command and the trajectory points will be sent to a control computer running ROS that will compute the robot path and starts the robot motion.

The programming feature presented in Lotsaris et al. [111] enables the user to command the robot to move towards a specific location or workstation inside the warehouse. The difference is that, for the workstation feature, holographic buttons with pre-defined coordinates are displayed over the workstation so the robot is always sent to the same location, whether other locations inside the warehouse can be chosen by clicking to any point inside the shop floor. In both cases, the digital twin of the mobile platform and the robot arm appears in the user’s field of view at the designated final position, as well as a line indicating the robot’s planned path.

Lastly, Dimitropoulos et al. [115] developed two programming approaches. The first is a direct movement of the end-effector using manual guidance, where the operator grabs the dedicated handles installed at the gripper of the robot and moves the end effector to his preferred position. The second one is an indirect AR-based control of the end-effector position, through which the operator places a virtual 3D holographic end-effector at the desired position where the physical end-effector should move to.

#### 4.4.5. Applications—Quality Control

This section describes the usage of Augmented Reality as means of quality assurance in a human–robot collaborative task. In Bolano et al. [106], the developed system is capable of identifying defects on the manipulated parts, which needs to be inspected by the operator. To signal that human intervention is needed, the projection system casts a blinking marker over the respective piece, while, in Schmitt et al. [110], the system is capable of identifying if the parts are correctly positioned and if all screws have been inserted and signaling it to the user. If any of these situations is not correct, a notification is projected in the same area used for instructions.

### 4.5. User Interaction Modality

Most of the selected papers use gesture as the main interaction modality, as shown in Table 14 and Figure 18. This is because most systems used to superimpose the interface into the user field of view are also capable of tracking and recognizing gestures offering an easy and intuitive way for manipulating the augmented environment while maintaining focus on the task.

Gesture recognition is the process of recognizing meaningful human movements that contain useful information in human–human or human–robot interaction. Gestures can be performed by different body parts and combinations thereof, such as hands, head, legs, full-body, or upper body. Hand gestures have received particular attention in gesture recognition. Hand movement provides the opportunity for a wide range of meaningful gestures, which is convenient for quickly and naturally conveying information in vision-based interfaces [121].

Speech recognition is the process of recognizing and appropriately responding to the meaning embedded within the human speech. By allowing data and commands to be entered into a computer without the need for typing, computer understanding of naturally spoken languages frees human hands for other tasks [122].

Gaze-based interaction can be split into four categories, namely: Diagnostic, normally used for assessing one’s attention towards a task, which is mainly performed offline following its recording during the performance of some task; Active, normally used for command effects, which uses real-time data provided by eye trackers such as *x* and *y*-coordinates and blink inputs; Passive, which usually does not imply any specific user action, however, implies a change to the display in response to the eye movement; and Expressive, which reflects the user’s eye movement and implicit expressions, normally used for mimicking realistic gaze behavior [123].

A graphical user interface is a collection of graphical components that enables information exchange between the user and a computer program, such as icons, buttons, text boxes, and menus. These components can be interactive, accepting inputs through mice, keyboards, and touch interfaces, or not [124,125].

A tangible user interface (TUI) can be defined as an interface where everyday physical objects play a central role as both physical representations and controls for digital information. These physical objects can translate user actions into input events in the computer interface through tactile manipulation and physical expressiveness by coupling digital information with physical objects and environments [126,127].

Works that make use of projection and head-mounted displays have a clear preference for gesture recognition as an input method since it is the most seamless way of manipulating 3D holograms. Through gestures like air tap, tap and hold and scrolling among others, the user can select, press, move, drag, resize and rotate holograms, as well as navigate through digitally displayed interfaces.

Some works demonstrate a preference for speech input methods arguing that voice commands for actions like passing to the next instruction or acknowledging the conclusion of a task as mean to not occupy the operator’s hands for interacting during the task, such as Andronas et al. [113].

Paletta et al. [103] and Chan et al. [88] make usage of diagnostic and active gaze inputs, respectively. This means that, on the first work, eye movement and gaze tracking were used for assessing the operator’s concentration level, and task switch rate, while on the second work, the user could interact with the holograms or give input to the system by staring at some point of interest or blinking.

Papers that make use of Hand-Held or external devices, such as smartwatches, enable the user to interact with the augmented environment through the use of GUI components such as buttons, icons, text boxes, and menus that activate or deactivate numerous different functions. For example, in Makris et al. [92], Michalos et al. [91], Gkournelos et al. [98] and Michalos et al. [99], the developed GUI interface can provide, upon user request, shop floor status, production data and enable and disable safety areas visualization.

Lastly, Materna et al. [89] is the only paper that makes use of a TUI, using a touch-enabled table to provide an input method for the operator.

One interesting fact that is worth being mentioned is that the three papers that did not present any user input modality (Bolano et al. [106], Hald et al. [108] and Vogel et al. [109]) are projector based applications. This may be due to the researcher considering that the implementation of interaction methods on those applications would not contribute to the aggregated value of the proposed research.

### 4.6. Visualization Methods

**Figure 19:**
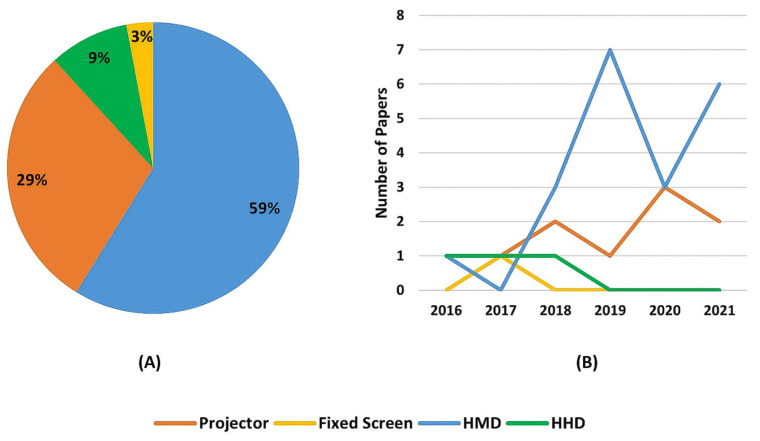
(**A**) Percentage of visualization methods; (**B**) user interface usage over time.

Most of the selected papers focus employ HMDs (59%) and projectors (29%) to convey the digitally generated content, Figure 19A. Moreover, Figure 19B shows, from 2017, an increasing tendency of usage towards HMD and projector-based interfaces, while HHDs and Fixed screens have fallen into disuse. This could have been expected since the first two technologies are capable of enhancing the user’s environmental perspective and situational awareness without occupying their hands during task execution and without demanding an attention shift from the task being performed to an instruction screen or paper, as discussed in Table 1. Moreover, the current state-of-the-art HMDs are compactly imbued with plenty of advanced technological software and hardware that ease their application in industrial environments, while also allowing operators freedom of movement and mobility throughout the entire shop floor.

**Figure 20:**
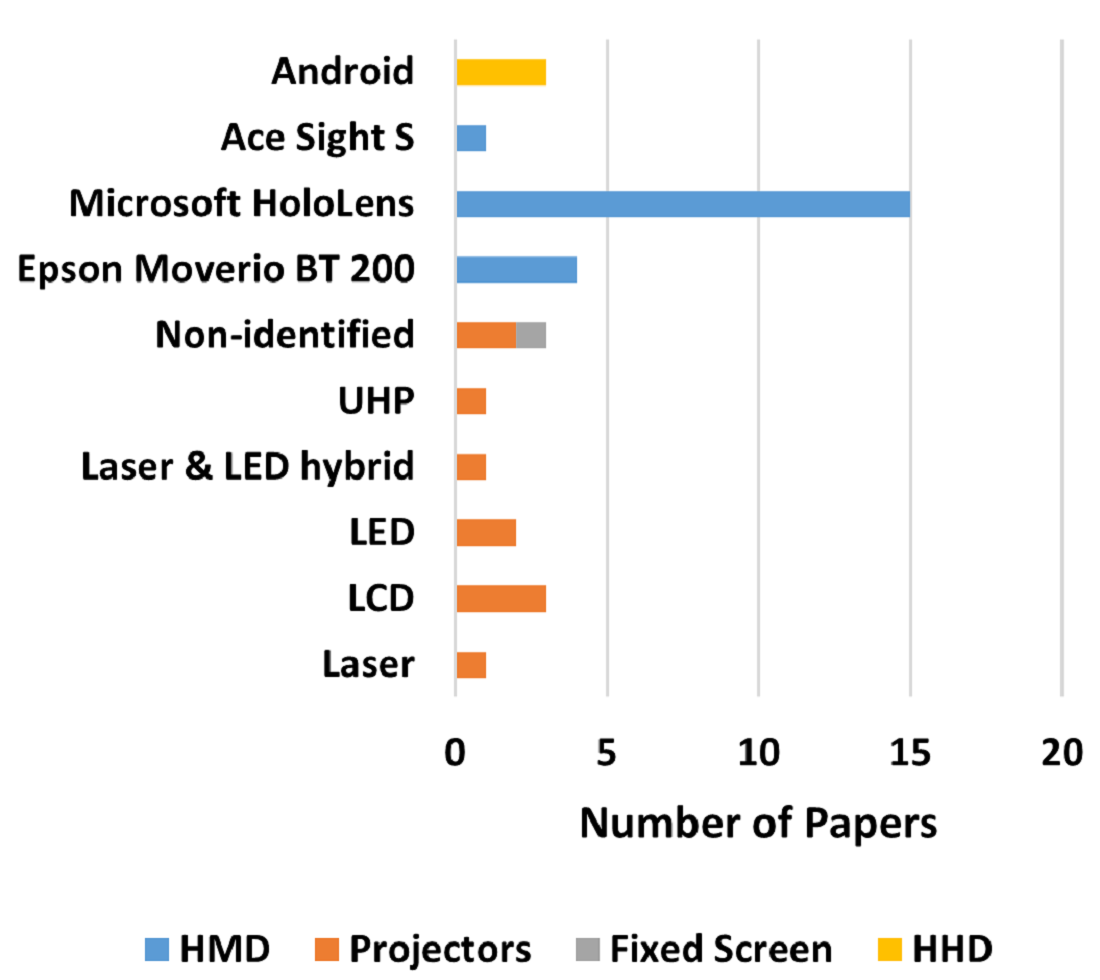
Equipment by visualization technology.

For example, the most used equipment among the selected papers, Microsoft HoloLens [56], as seen in Figure 20, comprises a set of visible light and infrared cameras, a depth sensor, an inertial measurement unity (accelerometer, gyroscope, and magnetometer), an image capturing camera, a microphone array, and integrated speakers, alongside other connectivity and computing hardware. By taking advantage of the mentioned hardware in combination with built-in software, the HoloLens 2, provides environment understanding features, such as six degrees of freedom positional tracking and spatial mapping; and human understanding features, such as hand and eye-tracking and voice control—enabling then the operator to interact with holograms virtually superimposed in his field of view and connect to other available equipment, or in other words, insert the human in the loop.

Regarding HHDs, android based equipment is preferred over other operational systems mostly due to its capability to be easily integrated into numerous devices, applications, and application programming interfaces (APIs). The same is true for works that used other wearable devices, such as smartwatches, for providing a GUI to the operator.

Concerning projector-based approaches, even though some projection aspects such as brightness, resolution, and contrast are critical factors that must be considered when choosing a projector for a specific application, the projection method does not demonstrate to exert much influence on the researcher’s decision of which projector to use, since no clear preference is shown towards a specific approach. The selected papers lead towards a faint tendency towards LCD and LED projectors.

### 4.7. Tracking Methods

**Figure 21:**
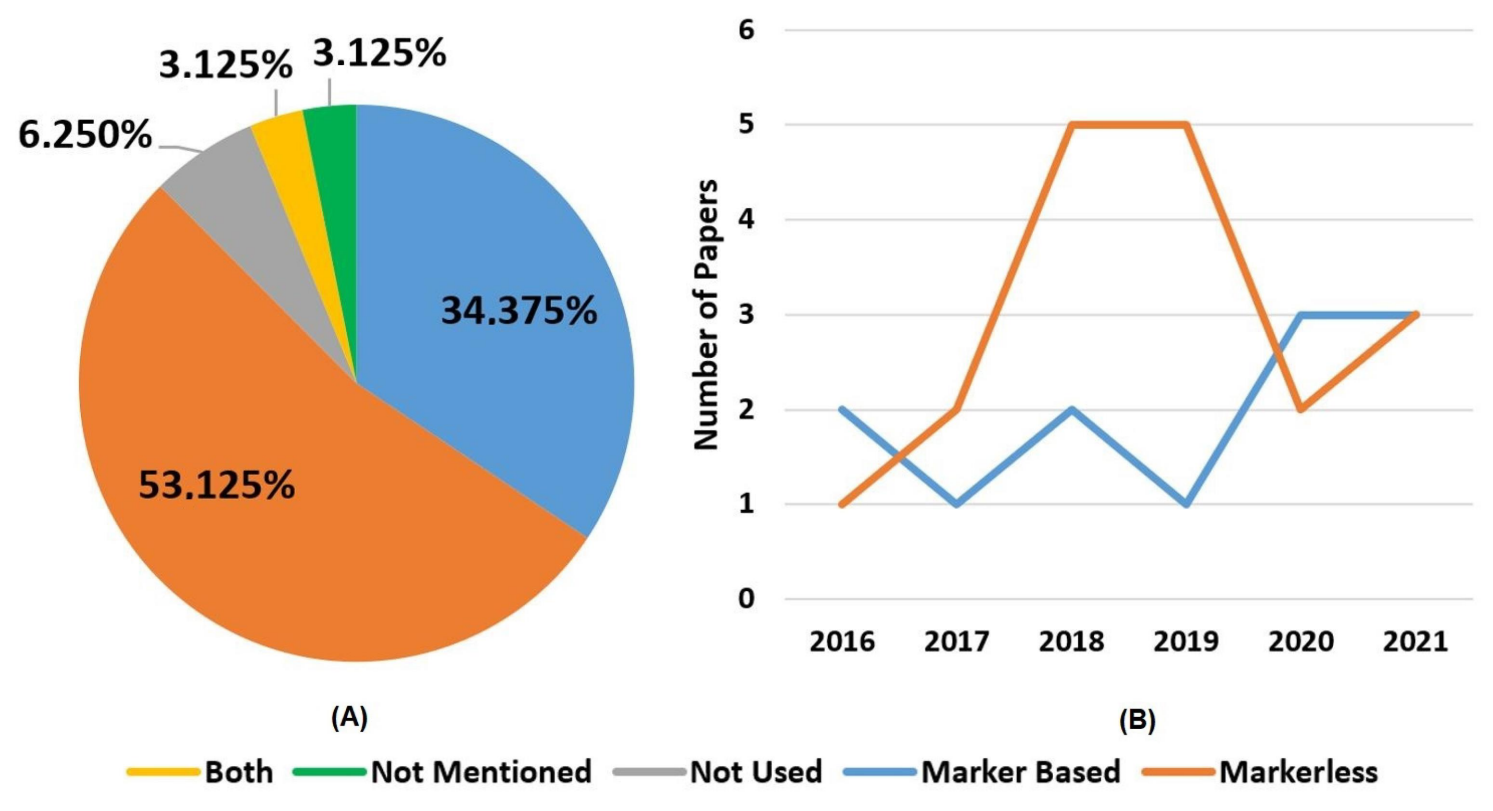
(**A**) Tracking methods over time; (**B**) tracking methodology by papers.

**Figure 22:**
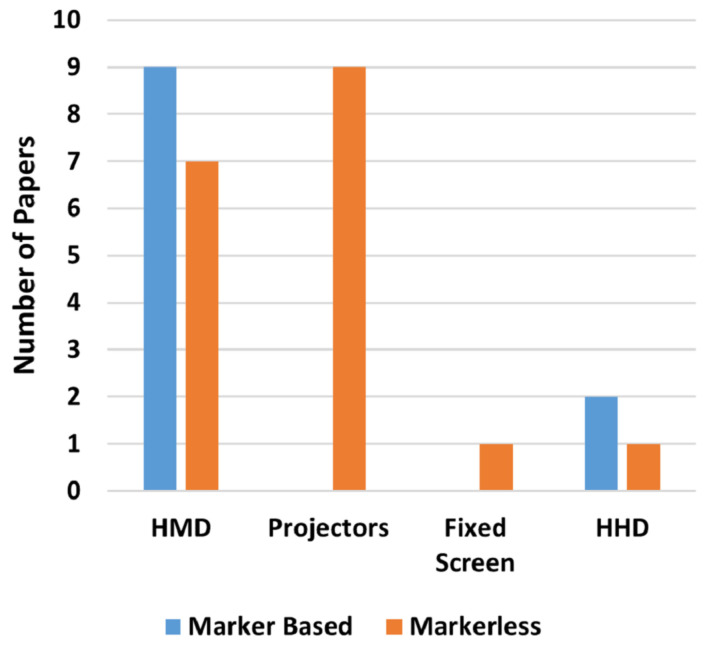
Tracking methodology by visualization technology.

Regarding tracking methods, even though marker-based approaches are showing an increasing usage tendency, there is still a preference towards markerless approaches over the marker-based ones, as can be seen in Figure 21A,B. This is mainly because of projection systems, Figure 22, which are currently focusing on feature extraction software for tracking object positions. Even though some may use markers for a first calibration, once done, they hardly need to be re-calibrated since they are not mobile. Moreover, the combination of external tracking hardware, such as depth and RGB cameras, with machines imbued with great computational power, allows run time comparison of the captured frames against stored CAD files.

Even though HMDs and HHDs do not have as much built-in computational power, most already have a built-in camera. Since this equipment allows the user to walk around the shop floor, the hologram may deviate from the original anchor point, making the usage of markers more interesting for a fast re-calibration. Another argument that may play in favor of markers, in this case, is the fact that built-in cameras normally do not have the same quality for image capturing when compared to external cameras that are proper for that.

On the other hand, implementing feature tracking to these systems is not impossible, since they have a reasonable computational processing power. Moreover, markerless systems are also handy for applications where physical markers would suffer due to poor environmental conditions or are extremely dynamic, as explained in Section 1, which may favor the use of markerless approaches over time.

### 4.8. Research Type and Evaluation Methods

Selected papers are equally distributed between the experimental (43.75%) and case study (43.75%) research types, while concept papers (12.50%) do not have as much presence, as can be seen on the research type column of Table 12. From this, it is possible to infer that the usage of augmented reality for human–robot collaboration is passing the birth phase and obtaining momentum on the maturation phase, thus not being considered a consolidated research field yet.

**Figure 23:**
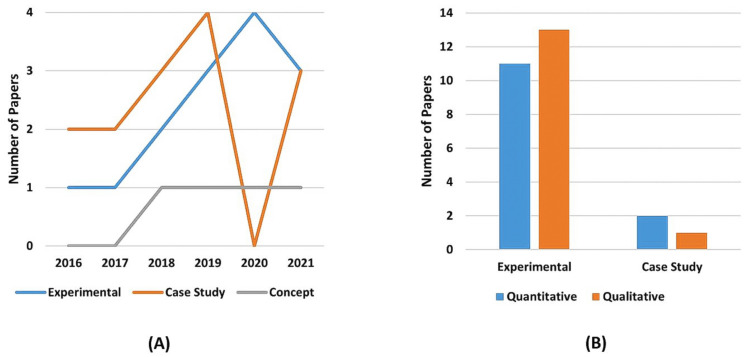
(**A**) Evaluation methodology by research type; (**B**) type of study over time.

As seen before, in Figure 14, this is a growing research field where the potential of applying augmented reality as a tool to enhance the operator in human–robot cooperative and collaborative tasks is still being investigated. This is corroborated by the fact that qualitative results are still being more analyzed than quantitative results in experimental papers, Figure 23B, which may indicate some uncertainty about the usage of Augmented Reality wearable equipment, especially in terms of usability and ergonomics. Moreover, most of the experimental research found is still being tested on a laboratory scale, not yet reaching real production sites.

It is clear that the most used qualitative measurement method was usability questionnaires, especially the System Usability Scale (SUS) questionnaire for evaluating the system usability (26.66% of the works), and the NASA-Task Load Index questionnaire for assessing participants cognitive load (26.66% of the works), as can be inferred from the second column of Table 15. Customized questionnaires are also used by 40% of the research. They are used for measuring several subjective aspects, such as safety feelings, trust, and task load, without the usage of a validated methodology. On the quantitative side, the most used measurement was the task execution time for assessing performance improvements (69.23% of the works) and the Rapid Upper Limb Assessment (RULA) technique for evaluating ergonomics (15.38% of the works).

As can be depicted from Figure 23A, there is an increasing tendency towards experimental studies. Moreover, the case study research type follows the same upward trend, despite the 2020 volatility. On the other hand, concept studies have been steadier throughout the analyzed years. Due to the curve fluctuation of the case study and the limited sample size, it is hard to draw any conclusions about the growth rate regarding the studies types, even though it may suggest a steeper growth for the experimental and case study types over the concept papers.

Even though concept studies provide interesting development ideas, researchers do not show much focus on this type of research. This apparent lack of interest in concept papers shows that researchers are more interested in achieving tangible results that can corroborate and prove the advantages of applying the developed application or new technologies to different areas.

### 4.9. Review Results

Although it is not a consensus, most works demonstrate that AR assistance improves the operation efficiency. Andersen et al. [93] shows that the time spent to complete the task was almost identical for all his experimental approaches (projector, fixed screen and text baseline), since the majority of the total time was taken up by the robot’s movements and not by the actions of the participants. Chan et al. [88] reported that their HMD-based AR system promoted a faster task execution than the no assistance baseline and the two joystick assistance trials. The quantitative results provided in Hietanen et al. [51] also shows that both the projector and HMD Augmented Reality approach performed better than the no assistance baseline, where the robot was not moving in the same workspace as the operator. This difference can be explained by the robot idle time, which is much lower for the two AR-based approaches. The difference between the HMD and the projector system is marginal. In Kalpagam et al. [49], the projection case presented significantly better performance when compared to the mobile and the text baseline. In the Tsamis et al. [90] experiments, the reduction in the task completion time is reportedly related to a reduction in the robot’s idle time: since the users were able to see both the current and planned state of the robot, they could avoid entering the robot’s workspace, thus not triggering the safety stop. Comparing the overall performance between an experimental and a control group, though, Paletta et al. [103] found that using the HMD AR assistance system implied a slight increase in completion time. The way instructions are presented to the user may significantly impact the system’s efficiency. As demonstrated by Materna et al. [89], participants took a considerable amount of time to understand how to perform the requested task, especially for the case of repeating sequences.

Concerning production cycle times, the quantitative results of the works assessed indicate that most operators managed to achieve a faster cycle time by using the application developed, Dimitropoulos et al. [115]. Michalos et al. [99] showed that the change from a manual operation to an Augmented Reality assisted human–robot collaborative scenario was able to reduce the production cycle time by almost 36%. Similarly, by changing the manual assembly scenario to an AR-assisted human–robot collaborative one, Papanastasiou et al. [105] promoted an increase in the operator average saturation level from 88% to 97%, and reduced the workstation cycle time from 45 s to 42 s.

Effectiveness was another metric that reportedly presented significant improvement in most experimental results. Andersen et al. [93] show that the projector-based approach had better performance than the fixed screen and text baseline approach, presenting no need for help intervention or wrong actions performed by the user. In its turn, Kalpagam et al. [49] measured effectiveness through the percentage and accuracy of task completion, where the projection system helped to improve the percentage of task completion and to lower the number of errors during the execution. In terms of task understanding time, measured as the time spent by the participant to read or look at the instructions, the projection-based approach demanded less time than other baseline systems. Moreover, the standard error for all subtasks using the projected mode was significantly lower than the printed and mobile display, implying that most participants took a similar amount of time to understand a subtask. The results presented by De Franco et al. [50] suggest that the subjects performed the polishing task more accurately using a feedback interface. The trial with the AR HMD feedback showed the lowest standard deviation of the applied force, implying that the users were able to keep an almost constant effort throughout the task, and therefore was considered the best feedback method for the polishing application. Even though Danielsson et al. [48] employed an AR-based assistance system, inexperienced users were not able to perform error free assemblies. Authors point out that this outcome might be related to the implementation of their system, and how the instructions were presented to the user.

All studies that measured the user’s ergonomics described improvement with respect to the baseline. The MURI analysis result presented by Michalos et al. [99] showed an improvement of almost 40% in the operator’s movement. In Dimitropoulos et al. [115], the RULA evaluation showed that the system improved the maximum ergonomic values for 4 out of the 5 operators. The RULA evaluation performed in Wang et al. [107], in turn, indicated that the distribution of the tasks between the robot and the operator have a significant impact on the operator’s ergonomics.

Other metrics used to evaluate the developed systems were the mean concentration level, amount of robot utilization, and the operator’s proximity to the robot. Regarding the mean concentration level, Paletta et al. [103] found that the group with AR assistance presented significantly above the concentration level for the group without the AR assistance system. Moreover, the expected time for the next task switch significantly decreased for the group with assistance. Concerning the amount of robot utilization, in Chan et al. [88], when able to choose the robot path, participants using the AR system were more likely to use the robot than the participants using the joystick approach. This exemplifies that, even when the technology is capable if the interface to the technology is lacking, users may prefer not to use it. Finally, Hald et al. [108] could not draw obvious conclusions or tendencies from the participant’s proximity to the robot and GSR measurements.

All studies that qualitatively evaluated the usability of the developed AR systems reported promising user acceptance towards the proposed approaches. Tsamis et al. [90] obtained above-average System Usability Scale (SUS) questionnaire results regarding the presented HMD AR system. Participants evaluated the system with higher scores for positively-worded questions related to ease of use and lower scores for negatively-worded queries related to system complexity. In Andronas et al. [113] and Materna et al. [89], the subjective measures also showed that the participants positively rated the usability of the developed application. Even though one of the test groups presented a negative tendency towards the developed application in Danielsson et al. [48], the remaining three groups positively evaluated the usability of the proposed system, resulting in a final overall positive evaluation of the system.

Although it is not a consensus for every NASA-TLX measured aspect, most works demonstrate that the use of AR assistance systems significantly reduce the operator’s perceived workload. In Materna et al. [89], subjective measures show that the participants positively rated the developed application regarding task load. The NASA-Raw Task Load Index (NASA-RTLX) questionnaire results obtained in Paletta et al. [103] demonstrate that those who used the AR assistance experienced significantly reduced levels of mental workload, time pressure, tension, and stress. In Chan et al. [88], the NASA-TLX questionnaire shows that the AR approach with the path choosing condition yielded the lowest physical demand, temporal demand, effort, and frustration. The AR approach with a predefined path registered the highest performance, and the no assistance condition demonstrated the lowest mental load. Wang et al. [107] results suggest that a task distribution that avoids frequent operation switches between the robot and the operator, reducing the human cognitive demand and constant contact with the robot, enables the operator to complete tasks more comfortably, and reduces the psychological load level.

Different personalized questionnaires were employed to measure several distinct metrics to evaluate the developed systems. Based on them, Hald et al. [108] deducted that unforeseen changes in robot movement speed affect user’s reported trust, but only for increases in velocity, where a decrease was identified, whereas the ability to hear the robot motors yielded no significant effect. Regarding information visualization, perceived user safety, and feeling of trust towards the robot, Tsamis et al. [90] showed that the AR system significantly outperformed the baseline solution, even though the HoloLens may offer limited ergonomics due to its weight and a narrow field of view. In Hietanen et al. [51], the qualitative results show that the projector-based approach was considered the safest, whereas the HMD was the unsafest by a clear margin. The amount of information needed to understand the task was smaller for the baseline approach, while the HMD was the most difficult to understand. Ergonomic-wise AR assisted systems were superior to the baseline approach, with the projector-based system being considered the best. The same is true for the competence or self-performance evaluation. Results obtained from the applied questionnaires, in De Franco et al. [50], showed that, for the user, it was easier to keep the focus on the task execution using the AR HMD approach and that it also had the best force indicator feedback. Participants also indicated that, even though it introduced an improvement in the perception of the applied force when compared to the no-feedback trial, using the fixed screen feedback was the most tiring and least easy to perform. Overall, the participants felt satisfied with the proposed AR interface and the amount of psychological effort required. Through the customized Likert scale applied in Lamon et al. [100], participants stated that the task execution using the collaborative setup with an AR device requires less physical and psychological effort than the manual approach. This perception led the participants to feel satisfied with the proposed collaborative system, while the manual setup task was perceived to be harder to perform. The participants also stated that understanding the action to be performed while keeping the focus on the task execution was effortless with the aid of the AR interface. Therefore, they agreed that the worker could carry out the same task for a long time with the AR setup and that collaborative robots could help workers to improve task performance. In Kalpagam et al. [49], the projection system was perceived to be better regarding human–robot fluency, clarity, and feedback. The participants also favored projection and mobile display conditions regarding task execution, human–robot collaboration, and attitude. Lastly, in Dimitropoulos et al. [115], all subjects indicated above-average satisfaction with the developed technology.

Other techniques employed to evaluate the proposed approaches were the after scenario questionnaire (ASQ), the Situation Awareness Rating Technique (SART), verbal feedback, and user free additional comments. The ASQ questionnaire applied in Andersen et al. [93] showed that regarding satisfaction, ease of usage, and support information, the text baseline was considered the worst approach. The projector was considered the best approach, being slightly better for ease of use and considerably better regarding support information when compared to the fixed screen approach. The participants also pointed out that the text approach gave an overview of the current state of the overall task and knew what the robot intended actions beforehand, which they considered would also be advantageous to have in the fixed screen and projection approach. The results of the (SART) questionnaire, in Paletta et al. [103], depict a significant increase in understanding, support of attention, and situational awareness dimensions, and a decrease in attention demand for the group using the AR assistance application. The verbal feedback received in Bolano et al. [106] indicated that the robot’s target and motion projected AR information was intuitive and understandable. It was also reported that the visualization of the robot swept volume on the worktable turned out to be handy to quickly understand the area where the robot was not planning to move during the execution of its current motion. In addition, the Tool Center Point (TCP) trajectory representation was more suitable for interpreting the end-effector path, which is very relevant for some tool types. Therefore, the presented information enabled the users to have more comfort and confidence in the interaction and decreased the anxiety caused by the lack of feedback from the robot.

Finally, the additional comments gathered in Chan et al. [88] referred to the AR assistance system as being the best and fastest one. On the other hand, the joystick approach was more accurate. Moreover, in Andronas et al. [113], participants expressed that the personalization of the system was very convenient and that it did not require advanced skills for using the system, being easy to get familiar with the controls and information. Furthermore, some participants also stated that the interface helped them in certain aspects of operation training, such as reducing errors and adaptation to working with robots in a fenceless environment. In Hietanen et al. [51], free user comments highlighted that the HMD augmented information blocks, to some extent, the operators’ field of view. Moreover, according to the author, the employed device is quite heavy, which can create discomfort and decrease the feeling of safety. Kalpagam et al. [49] showed, through the collected free-response data, that all participants favored the projected mode over the printed and mobile conditions. Major themes mentioned included ease of task performing, the ability to complete a task accurately, clarity of instructions, feedback supply, intuitiveness, system oversight, and, finally, perceived safety.

### 4.10. Future Developments

Some of the analyzed papers bring to light interesting insights when proposing future developments for the continuity of their work. From those, it is possible to derive the next steps in the Augmented Reality for Human–Robot Collaboration and Cooperation research field.

Makris et al. [92] highlights the need for testing AR applications in industrial environments, stating that applications do not yet get to industrial environments staying as small-scale laboratory experiments. Assessing their impact and validation in real working processes, which can significantly differ from small-scale laboratory experimental results, can provide insights into whether laboratory experiment results realistically reflect results that can be generalized to the industry.

Makris et al. [92] also states that the usability of markerless approaches for dynamic HRC workspaces needs to be further studied and compared to the current marker-based ones to achieve relevant insights to determine to which conditions each approach would be a better fit. Corroborating with this idea Michalos et al. [91] proposes on top of that further studies on tracking algorithms fine-tuning for preventing holograms vibration and jitters, due to constant re-positioning that may be caused by environmental issues, or the quality of recognition devices, which may cause discomfort to the user when using the application for long periods.

Emphasizing the fact that HHDs are not adequate for HRC context, occupying the operators’ hands and demanding focus changing for checking info and then proceeding to the task, wasting time, Michalos et al. [91] also suggests that future works should focus on the HMDs technologies, to assess if the ergonomic and performance negative effects of applying HHD devices could be mitigated by the change of augmented device technology.

Moreover, Michalos et al. [91], Liu and Wang [94] and Schmitt et al. [110] state that some emphasis should be applied towards determining standards and guidelines for augmented reality support systems and interface development to make it more user-friendly and ergonomically appealing to the user. Assessing which and how much information should be displayed to the user and in which conditions (color, size, transparency level …) to not overload the user’s field of view and increase the operator cognitive load. Corroborating the suggestion on establishing standards for developing augmented reality support systems for operators, Danielsson et al. [48] point towards comparing the usage of animated instructions over static ones.

Still, on the standardization topic, but a little further from the previous suggestions, Wang et al. [107] advises that there is also a need to do more research on human factors for designing HRC workstations.

Danielsson et al. [48] also expresses interest in studies assessing how AR affects focus and concentration levels, as studied by Paletta et al. [103], which also shows interest in delving deeper into this subject. Not far from this idea, Argyrou et al. [96], to improve task monitoring status through the fusion of robot and human data, highlight a research line towards the use of AR technologies for gathering human data.

Andersen et al. [93] advocates in favor of testing proposed approaches with a larger pool of participants to obtain more statistically significant conclusions. Moreover, Kalpagam et al. [49] complements this idea by encouraging tests with additional participant groups, including non-engineers, individuals with prior line manufacturing experience, and individuals representing a broader age range, since the experiments were done only with undergraduate and graduate engineering students cannot be generalized for a broader group.

Another field of study that already is being explored but still shows room for improvements, according to Gkournelos et al. [98] and Ji et al. [101], is multi-modal interfaces, such as those discussed in Gkournelos et al. [98] and Andronas et al. [113] for example. Moreover, Ji et al. [101] also draws attention to multi-modality application towards safety assurance, which is also outlined by other papers such as Michalos et al. [99] and Papanastasiou et al. [105]. The fact that there are few, or no AR-certified safety approaches makes it a prominent area. Papanastasiou et al. [105] emphasizes that human trust in manufacturing robots and the safety of the solution must be further assessed directly in industrial environments to have a better perception of the level of acceptance for the solution.

Usability of the proposed approach also needs to be studied since, according to Papanastasiou et al. [105], the use of wearable devices (HMDs, smartwatches) might find resistance between operators for their adoption, so testing usability with the target audience is important, ratifying Kalpagam et al. [49]. Furthermore, Bolano et al. [106] and Chan et al. [88] instigate the assessment of feedback channels for bidirectional human–robot communication such as gestures, haptic devices, and speech recognition to improve user experience and performance.

According to Lamon et al. [100], De Franco et al. [50] and Hietanen et al. [51], task allocation is a general problem and has been discussed intensively in other fields, while less effort has been devoted in industrial scenarios, involving mixed human–robot teams and, in particular, to the factors that should be considered in allocating tasks among a heterogeneous set of agents in collaborative manufacturing scenarios. Therefore, future works may focus on the coordination and allocation of multiple human–robot teams for different required tasks. Complementing this idea, Tsamis et al. [90] demonstrate the necessity of applying multi-user systems, enabling operators to cooperate among themselves.

Some authors such as Hald et al. [108] and Andronas et al. [113] also speak in favor of assessing different methodologies for measuring and evaluating physiological and psychological aspects. Ergonomics, concentration level, stress, and trust, among other human aspects, are currently assessed through short period experiments. Hald et al. [108] states that, with a longer experiment, it would be interesting to look into the recovery in trust. The same can be applied for other aspects, to understand if there will be a behavior change after a long period of technology usage, as the users will adapt over time to certain conditions.

Lotsaris et al. [111] and Andronas et al. [113] speak in favor of developing flexible, adaptable, and scalable approaches, towards lowering the amount of time and effort needed for the deployment of the application. Lastly, Andronas et al. [113], Dimitropoulos et al. [114] and Dimitropoulos et al. [115] propose studies for improving object and human actions detection using the wearable headset and other wearable devices such as smartwatches.

## 5. Conclusions

This paper presents the current state of the art in the use of augmented reality for human–robot collaboration and cooperation in the industrial context. To do so, a systematic literature review of 387 papers, where 32 were deemed relevant, was conducted. The SLR research protocol was based on the SALSA framework and inspired by similar reviews (De Pace et al. [4], Fernández del Amo et al. [78]). It comprises the analysis of the selected papers regarding several topics, such as application field, used robots, user input mechanisms, visualization and tracking methods, research type, evaluation techniques, demographic, publication, and terminology subjects. The result of this analysis was used to answer the three research questions: (Q1) What are the main AR visualization technologies used in industrial Human–Robot collaboration and cooperation context? (Q2) What are the main field of application of AR in industrial Human–Robot collaboration and cooperation context? and (Q3) What is the current state of the art of AR applications for Human–Robot collaboration and cooperation? Is research focusing on experimental or concept applications? What are the most used assessment techniques and indicators? What are the Research gaps presented in AR for industrial Human–Robot collaboration and cooperation context?

The first major contribution of this paper is the identification of the main AR visualization technologies used in industrial human–robot collaboration and cooperation context (research question Q1, Section 3.1). HMD interfaces have gained relevance since 2018, followed by a not as much exponential interest growth of projected interfaces (Section 4.6). On the other hand, Fixed Screen and HHD interfaces have fallen into disuse for HRC applications in the past three years. As mentioned by De Pace et al. [4], these results are not unexpected: HMD and projector-based interfaces are hands-free, which enable greater operator mobility within the industrial warehouse and does not require as much attention shift as Fixed Screen and HHD approaches.

It is acceptable to assume that these characteristics would allure researchers to focus new studies on these two interfaces, especially on HMD. Moreover, since newer headsets are capable of performing scene understanding and natural gesture interactions without the need of external devices, it is expected that the research interest of this devices quickly grows. The same argument is valid when comparing the Augmented Reality glasses. Neither the Epson MOVERIO [57] nor the Acesight S [128] do not provide the same capabilities as the Microsoft HoloLens [56], which explains the researchers’ preference for using the Microsoft device. Therefore, the exponential growth of studies involving HMDs can also be related to the first version of the Microsoft HoloLens in 2016.

Comparing the projector types used for Human–Robot collaboration, there is not a clear preference towards a particular projection method (Section 4.6). Even though brightness, resolution, contrast, and other factors must be taken into consideration when choosing a projector, the selected papers lead to a faint tendency towards LCD and LED approaches.

Although all HHDs were based on Android, it is not possible to assume this operating system as a preference due to the small sample size. Some factors that may justify the inclination of researchers towards Android are the potentially lower cost, the larger number of platforms and devices, the easier adoption for developers, and the APIs to take full advantage of the embedded sensors.

Concerning tracking methods, there is a tendency to use markerless approaches (Section 4.7). That is mostly driven by projection systems, which use feature extraction software for tracking object positions. However, marker-based approaches are gaining strength, especially due to the HMDs. The straightforward implementation of marker-based approaches and the lower computational power required might be the reasons that sustain this increasing tendency. On the other hand, the new Augmented Reality HMDs have a reasonable computational power and numerous sensors and cameras for spatial mapping, which may favor the use of markerless approaches over time.

The second major contribution of this paper is the identification of the main fields of application of AR in industrial human–robot collaboration and cooperation context (research question Q2, Section 3.1). The use of augmented reality for improving the operator’s awareness, trust, and safety feeling towards the robot is a constant concern, making the safety category the most researched one (Section 4.4). Since the operator needs to share the workspace with one or more robots, any additional layer of safety may help improve the operator’s condition and feeling towards the robot and the collaborative scenario. Although no Augmented reality safety approaches are yet certified, some of them have been showing promising results as active monitoring systems.

The capacity of augmented reality to display real-time information to the user field of view is essential to insert the human in the loop. Providing production live status, graphics, simulations, analysis and other operation-related information in a fast and intuitive way enables the user to be more assertive when making a decision that can affect the whole production line, or responding to emergencies.

Moreover, by showing to the user step-by-step text, images, videos, 2D or 3D representation of a workpiece, or animated instructions, augmented reality is vastly used for guidance applications. The main contributions for guidance applications are improving the operators’ performance by reducing the task completion time and abstracting the user of the necessity of memorizing numerous instructions for a variety of products, lowering their cognitive load, and enabling an untrained operator to be able to conduct any operation if needed. Augmented reality is also being vastly used for programming applications. Most of the applications developed in this field fall under the Human–Robot Interaction category since no further collaboration happens among the parts after programming. Finally, quality control is an area that is starting to be combined with augmented reality and is expected to gain more relevance over time.

Regarding the economic activity, the automotive industry conducts the largest number of studies by a considerable amount. This may be because the automotive industry has always been a pioneer when comes to testing emerging technologies and especially to the fact that it presents numerous challenges that are a perfect fit for evaluating different applications, from a smaller to a larger scale. Lastly, again by a large difference, assembly operations are the most studied activity when it comes to augmented reality applied for human–robot collaboration and cooperation.

The third major contribution of this paper consists of the identification of the current state of the art on AR applications for Human–Robot collaboration and cooperation (research question Q2, Section 3.1). Referring back to Section 4.8, it is possible to infer that qualitative evaluations are currently more employed than quantitative evaluations. It indicates some uncertainty about the usage of Augmented Reality wearable equipment, especially on human–centered perspectives, such as usability and ergonomics terms. The most used evaluation techniques are the System Usability Scale (SUS) questionnaire, for evaluating the system usability, and the NASA-Task Load Index (NASA-TLX) questionnaire, for assessing the participants’ cognitive load. On quantitative evaluations, the most used measurement is the task execution time, for assessing performance improvements, and the Rapid Upper Limb Assessment (RULA) technique, for evaluating ergonomic aspects.

AR applications demonstrated the ability to improve operators’ performance, accuracy, task understanding, safety feeling, and task awareness when compared to baseline assistance methods, such as printed and fixed screen based instructions. Moreover, the change of purely manual tasks to an AR-assisted collaborative scenario was demonstrated to reduce the production cycle time and improve the operator’s ergonomics. Concerning visualization, HMDs are deferred due to hardware aspects such as narrow field of view, occlusion, and weight, which might exert some influence on the operator’s safety feeling.

Industries will soon employ AR applications for a variety of cooperative and collaborative tasks. Therefore, future developments should rely on end-user experience and feedback to further improve human–robot collaboration features. Moreover, multi-agent communication and interaction between different operators, devices, and services should be explored to promote more dynamic information exchange throughout the industry. Thus, there is still much development and research to be done and questions to be answered before understanding and achieving the full potential of Augmented Reality on Human–Robot Collaboration and Cooperation.

## Figures and Tables

**Table 2:** Adopted SALSA framework.

Step	Outcome	Method
Protocol	Research Scope	PICOC framework
Search	Potential Data	Literature Search
Appraisal	Selected Studies	Selected Data Evaluation
Synthesis and analysis	Data Taxonomy	Data Features extraction and analysis

**Table 3:** Adopted PICOC framework.

Concept	Definition [76]	SLR Application
Population	The problem or situation the research is dealing with	Augmented Reality for human–robot cooperative and collaborative applications
Intervention	Existing techniques utilized to address the problem identified	Tools and techniques for the use of AR in collaborative tasks.
Comparison	Techniques to contrast the intervention against	Contrast between intervention techniques.
Outcome	The measure to assess the effect of the techniques applied to the population	Quantitative and Qualitative performance indicators.
Context	The particular settings or areas of the population	Augmented Reality and human–robot collaborative operations in the industrial domain.

**Table 4:** Exclusion criteria.

Type	Criteria	Statement
Exclusion	Index	Papers that are not indexed in a scientific publication venue
Exclusion	Year	Papers before 2016
Exclusion	Research Field	Papers that are not classified in search engines as engineering or computer sciences
Exclusion	Application Field	Papers that do not present industrial applications
Exclusion	Partial Coverage	Papers that do not present both AR and HRC
Exclusion	Scope	Papers that approach different and not related subjects
Exclusion	Gray, Secondary and Tertiary Literature	Thesis, reports, Reviews, books …
Exclusion	Short Papers	According to the categorization provided by the publication venue
Exclusion	Virtual Reality	Papers that use VR instead of AR
Exclusion	HRI only	Papers that present any kind of HRI that do not fit on the definition of human–robot collaboration or Cooperation

**Table 5:** Number of papers found per database.

Databases	N° of Papers
Scopus	93
Web of Science	91
ACM	72
IEEE Xplore	48
Ebsco	33
Dimensions.ai	27
ScienceDirect	14
Manual insertion	9

**Table 6:** Quality assessment criteria and statistical results.

ID	Criteria	Score Range
QC1	Normalized number of citations per year	0–1
QC2	Normalized Impact Factor	0–1
QC3	Does the article have all the keywords in the title?	0–1
QC4	Does the article present the current state of the art of AR in manufacturing applications?	0–1
QC5	Is the research objective clearly described?	0–1
QC6	Does the study identify the used hardware and software?	0–1
QC7	Is the methodology appropriate and detailed?	0–1
QC8	Are the results and conclusions appropriate and clear?	0–1
QC9	Does the article present an experiment to evaluate the application? If so, are the methodology and the evaluation criteria well described?	0–1
QC10	Do the authors describe the study limitations?	0–1

**Table 7:** Papers assessment and general information.

Paper	Country	Pub. Year	Publication Venue
Makris et al. [92]	Greece	2016	CIRP Annals Manufacturing Technology (CIRP Ann.—Manuf. Technol.)
Michalos et al. [91]	Greece	2016	Procedia CIRP
Andersen et al. [93]	Denmark	2016	IEEE International Conference on Robot & Human Interactive Communication (IEEE RO-MAN)
Liu and Wang [94]	Sweden	2017	Procedia Manufacturing
Danielsson et al. [48]	Sweden	2017	Procedia CIRP
Vogel et al. [95]	Germany	2017	Procedia Manufacturing
Argyrou et al. [96]	Greece	2018	Procedia CIRP
Lee et al. [97]	South Korea	2018	IFIP Advances in Information and Communication Technology (IFIP—AICT)
Gkournelos et al. [98]	Greece	2018	Procedia CIRP
Kalpagam et al. [49]	United States	2018	IEEE Robotics & Automation Magazine (IEEE Robot. Autom. Mag.)
Materna et al. [89]	China	2018	IEEE International Conference on Robot & Human Interactive Communication (IEEE RO-MAN)
Michalos et al. [99]	Greece	2018	Mechatronics
Lamon et al. [100]	Italy	2019	IEEE Robotics and Automation Letters (IEEE Robot. Autom. Lett.)
De Franco et al. [50]	Hungary	2019	IEEE International Work Conference on Bioinspired Intelligence (IEEE IWOBI)
Ji et al. [101]	China	2019	International Conference on Computers & Industrial Engineering (CIE)
Mueller et al. [102]	Germany	2019	SAE International Journal of Advances and Current Practices in Mobility
Paletta et al. [103]	Austria	2019	IEEE International Conference on Emerging technologies and Factory Automation (IEEE ETFA)
Kyjanek et al. [104]	Germany	2019	International Symposium on Automation and Robotics in Construction (ISARC)
Papanastasiou et al. [105]	Greece	2019	International Journal of Advanced Manufacturing Technology (Int. J. Adv. Manuf. Syst.)
Bolano et al. [106]	Germany	2019	IEEE International Conference on Robot & Human Interactive Communication (IEEE RO-MAN)
Chan et al. [88]	United States	2020	IEEE International Conference on Robot & Human Interactive Communication (IEEE IROS)
Hietanen et al. [51]	Finland	2020	Robotics and Computer-Integrated Manufacturing (Robot. Comput. Integr. Manuf.)
Wang et al. [107]	China	2020	Lecture Notes in Computer Science (LNCS)
Hald et al. [108]	United States	2020	IEEE International Conference on Robot & Human Interactive Communication (IEEE IROS)
Vogel et al. [109]	Germany	2020	IEEE International Conference on Emerging technologies and Factory Automation (IEEE ETFA)
Schmitt et al. [110]	United States	2021	ACM/IEEE International Conference on Human–Robot Interaction (ACM/IEEE-HRI)
Lotsaris et al. [111]	Greece	2021	Procedia CIRP
Luipers and Richert [112]	Germany	2021	IEEE International Conference on Artificial Intelligence and Computer Applications (IEEE ICAICA)
Tsamis et al. [90]	Canada	2021	IEEE International Conference on Robot & Human Interactive Communication (IEEE RO-MAN)
Andronas et al. [113]	Greece	2021	Procedia Manufacturing
Dimitropoulos et al. [114]	Greece	2021	Procedia CIRP
Dimitropoulos et al. [115]	Greece	2021	Applied Sciences

**Table 8:** Papers’ quality evaluation.

Reference	QC1	QC2	QC3	QC4	QC5	QC6	QC7	QC8	QC9	QC10	Total
[51]	1	1	1	1	1	1	1	1	1	0	9.00
[49]	0.337	0.635	0.5	1	1	1	1	1	1	1	8.472
[88]	0.088	0.213	1	1	1	0.5	1	1	1	1	7.80
[89]	0.169	0.013	0.5	0.5	1	1	1	1	1	1	7.182
[91]	0.836	0.202	1	1	1	1	1	1	0	0	7.038
[90]	0	0.013	1	1	1	1	1	1	1	0	7.013
[104]	0.41	0.009	1	0.5	1	1	1	1	0	1	6.918
[92]	1	0.785	1	0	1	0.5	1	1	0	0	6.285
[48]	0.558	0.202	1	0	1	1	0.5	0	1	1	6.26
[107]	0	0.124	1	0.5	1	1	1	0.5	1	0	6.124
[99]	1	0.595	0.5	0	1	1	1	1	0	0	6.095
[50]	0.091	0	1	0	1	1	1	1	1	0	6.09
[115]	0.25	0.310	0.5	0	1	1	1	1	1	0	6.06
[97]	0.026	0.010	1	1	1	0.5	1	0.5	0	1	6.035
[105]	1	0.495	0.5	0	1	1	1	1	0	0	5.995
[108]	0.0175	0.213	0	0	1	0.5	1	1	1	1	5.73
[98]	0.467	0.202	0.5	0	1	0.5	1	1	0	1	5.67
[103]	0.11	0.014	0.5	0	1	1	1	1	1	0	5.627
[100]	0.477	0.601	0	0	1	0.5	1	1	1	0	5.578
[112]	0	0.006	1	0	1	1	1	1	0	0	5.00
[94]	1	0.026	1	1	0	0.5	0.5	0.5	0	0	4.525
[93]	0.403	0.013	0	0	1	0	1	1	1	0	4.416
[113]	0.25	0.026	0	0	1	1	0.5	0.5	1	0	4.275
[111]	1	0.202	0.5	0	0	1	1	0.5	0	0	4.202
[106]	0.18	0.013	0.5	1	1	0	1	0.5	0	0	4.195
[110]	0	0.025	0.5	1	1	0.5	1	0	0	0	4.025
[114]	0.25	0.202	0.5	0	1	0.5	0.5	1	0	0	3.95
[95]	0.79	0.026	0.5	0	1	0.5	1	0	0	0	3.816
[109]	0.0175	0.014	0.5	0	1	1	1	0	0	0	3.531
[101]	0.023	0	1	0.5	0	1	1	0	0	0	3.522
[96]	0.156	0.202	0.5	0	1	0.5	1	0	0	0	3.357
[102]	0.091	0	0	0	0	0	0.5	1	0	0	1.59
Mean	0.375	0.2	0.625	0.3475	0.875	0.734	0.922	0.7187	0.4375	0.25	5.481
Std. Dev.	0.374	0.263	0.36	0.448	0.336	0.335	0.184	0.400	0.504	0.44	1.620

**Table 9:** Search general information.

Reference	String Combination	Level of Interaction	Databases (O—Manually Found, X—Found by the Search String)						
			ACM	Dimensions	IEEE	WOS	ScienceDirect	Scopus	Ebsco
[92]	Collaboration Augmented Reality	Collaboration		O		X/O	O	X/O	
[91]		Collaboration		O		O	O	O	
[93]		Cooperation		O	O	O		O	
[94]	Collaboration Augmented Reality	Cooperation		O		X/O	O	X/O	
[48]	Collaboration Augmented Reality	Collaboration		O		X/O	O	X/O	
[95]		Cooperation		O		O	O	O	
[96]		Cooperation		O		O	O	O	
[97]	Collaboration Augmented Reality	Cooperation		X/O		X/O		X/O	
[98]	Cooperation Augmented Reality	Collaboration		O		X/O	X/O	X/O	
[49]	Collaboration Mixed Reality	Cooperation		O	X/O	O		X/O	O
[89]	Collaboration Augmented Reality	Cooperation		O	X/O	X/O		X/O	
[99]	Collaboration Augmented Reality	Collaboration		O		X/O	O	X/O	X/O
[100]		Collaboration		O	O	O		O	
[50]	Collaboration Augmented Reality	Collaboration		O	X/O			X/O	
[101]	Collaboration Augmented Reality	Collaboration						X/O	
[102]	Collaboration Mixed Reality	Collaboration		O				X/O	
[103]		Cooperation		O	O	O		O	
[104]	Collaboration Augmented Reality	Collaboration		O				X/O	
[105]	Collaboration Augmented Reality	Cooperation		O		X/O		X/O	X/O
[106]	Collaboration Augmented Reality	Cooperation		O	X/O	X/O		X/O	
[88]	Collaboration Augmented Reality	Collaboration		O	X/O	X/O		X/O	
[51]	Collaboration Augmented Reality	Collaboration		X/O		X/O	X/O	X/O	X/O
[107]	Collaboration Augmented Reality	Cooperation		X/O				X/O	
[108]	Collaboration Augmented Reality	Collaboration		O	X/O	X/O		X/O	
[109]	Collaboration Augmented Reality	Cooperation		X/O	X/O	X/O		X/O	
[110]	Collaboration Augmented Reality	Collaboration	O					X/O	
[111]	Collaboration Augmented Reality	Collaboration		O			O	X/O	
[112]	Collaboration Augmented Reality	Cooperation		O	X/O			X/O	
[90]	Collaboration Augmented Reality	Cooperation		O	X/O	X/O		X/O	
[113]	Collaboration Augmented Reality	Collaboration		O			X/O	X/O	
[114]	Collaboration Augmented Reality	Collaboration		O			O	X/O	
[115]	Collaboration Augmented Reality	Collaboration		O		O		X/O	

**Table 10:** Papers’ details.

Reference	Robot	Tacking Method	Visualization Method	Visualization Equipment
[92]	COMAU NJ 130	Marker Based	HMD	Epson Moverio BT 200
[91]	COMAU NJ 370	Marker Based	HHD	ASUS Transformer Prime TF 201
[93]	Universal Robots	Markerless	Projector	Non-identified Laser projector
[94]	Universal Robots	Markerless	Fixed Screen	Non-identified television
[48]	Universal Robots UR3	Marker Based	HHD	Nvidia Shield Tablet
[95]	Kuka iiwa 14	Markerless	Projector	Non-identified LED-DLP projector
[96]	Universal Robots UR10	Markerless	HMD	Microsoft HoloLens
[97]	Non-identified	Markerless	HHD	Non-identified but android based
[98]	COMAU NJ 130 COMAU Racer 7	Both	HMD	Epson Moverio BT 200
[49]	Universal Robots UR5	Markerless	Projector	Non-identified LCD projector
[89]	Willow Garage PR2	Markerless	Projector	Acer P6600 UHP-DLP projector
[99]	COMAU NJ 130	Marker Based	HMD	Epson Moverio BT 200
[100]	Franka Emika Panda	Not-used	HMD	Microsoft HoloLens
[50]	Franka Emika Panda	Not-used	HMD	Microsoft HoloLens
[101]	ABB IRB 1200	Marker Based	HMD	Microsoft HoloLens
[102]	Universal Robots	Markerless	HMD	Microsoft HoloLens
[103]	Kuka iiwa LBR 7	Markerless	HMD	Microsoft HoloLens
[104]	Kuka iiwa LBR	Markerless	HMD	Microsoft HoloLens
[105]	COMAU Racer	Markerless	HMD	Epson Moverio BT 200
[106]	Universal Robots	Markerless	Projector	Non-identified video projector
[88]	Kuka iiwa LBR 14	Marker Based	HMD	Microsoft HoloLens
[51]	Universal Robots UR5	Marker Based	HMD Projector	Microsoft HoloLens LCD Projector
[107]	Universal Robots	Marker Based	HMD	Acesight S
[108]	Rethink Robotics Sawyer	Markerless	Projector	Non-identified projector
[109]	Kuka KR-60-L45	Markerless	Projector	Casio XJ-V110W
[110]	Han’s Robots Elfin 5	Markerless	Projector	Ulixes A600
[111]	Universal Robots	Marker Based	HMD	Microsoft HoloLens
[112]	ABB YuMi	Non-identified	HMD	Microsoft HoloLens
[90]	Universal Robots UR10	Marker Based	HMD	Microsoft HoloLens
[113]	Universal Robots	Markerless	HMD	Microsoft HoloLens 2
[114]	COMAU AURA COMAU Racer 5	Markerless	HMD Projector	Microsoft HoloLens 1/2 LCD Projector
[115]	COMAU AURA	Marker Based	HMD	Microsoft HoloLens 2

**Table 11:** Number of Papers by county.

Country	Number of Papers
Greece	10
Germany	6
United States	4
China	3
Sweden	2
Austria	1
Canada	1
Denmark	1
Finland	1
Hungary	1
Italy	1
South Korea	1

**Table 12:** Papers’ application details.

Reference	Industry	Activity	Research Type	Presented Evaluation?
[92]	Automotive	Assembly car axle and rear wheel group	Case Study	No
[91]	Automotive	Assembly car axle and rear wheel group	Case Study	No
[93]	Automotive Any	Pick-and-Place a small box	Experimental	Yes
[94]	Automotive	Assembly car engine	Experimental	Yes
[48]	Toy industry	Assembly wooden car toy	Case Study	No
[95]	Mechanic	Assembly/Screwing a non identified part	Case Study	No
[96]	Automotive	Assembly Diesel engine Euro 6 turbocharger	Case Study	No
[97]	Mechanic Industrial equipment	Assembly electric engines	Concept	No
[98]	Automotive White Goods	Assembly car axle and rear wheel group Sealing a refrigerator	Case Study	No
[49]	Automotive	Assembly car door	Experimental	Yes
[89]	Furniture Masonry	Assembly wooden chairs	Experimental	Yes
[99]	Automotive	Assembly car axle and rear wheel group	Case Study	Yes
[100]	Mechanic	Assembly aluminium profile	Experimental	Yes
[50]	Mechanic	Assembly aluminium profile Polishing surfaces	Experimental	Yes
[101]	Mechanic	Assembly a non-identified part	Case Study	No
[102]	Aeronautic	Assembly/Riveting aircraft fuselage	Concept	No
[103]	Toy industry	Pick-and-Place tangram puzzle (toy problem)	Experimental	Yes
[104]	Masonry	Assembly plywood	Case Study	No
[105]	White Goods	Assembly/Sealing Refrigerator	Case Study	Yes
[106]	Mechanic	Assembly non-identified parts	Case Study	Yes
[88]	Aeronautic	Assembly/Pleating aircraft fuselage	Experimental	Yes
[51]	Automotive	Assembly diesel engine	Experimental	Yes
[107]	Automotive	Assembly car gearbox	Experimental	Yes
[108]	Art industry	Drawing (toy problem)	Experimental	Yes
[109]	Any	Pick-and-Place parts from one station to another	Concept	No
[110]	Toy industry	Assemble a truck toy	Case Study	No
[111]	Automotive	Assembly car suspensions	Experimental	Yes
[112]	Any	Handover/Pick-and-Place non-identified parts	Concept	No
[90]	Any	Pick-and-Place parts from one station to another	Experimental	Yes
[113]	Automotive	Assembly car powertrain	Experimental	Yes
[114]	Transport systems Mechanic	Assembly elevator and riveting aluminium panels	Case Study	No
[115]	Transport systems	Assembly elevator	Experimental	Yes

**Table 13:** Papers’ applications.

Reference	Application				
	Guidance	Feedback	Safety	Programming	Quality Control
[92]	X	X	X		
[91]	X	X	X		
[93]	X		X		
[94]	X				
[48]	X	X			
[95]		X	X		
[96]	X		X		
[97]		X			
[98]	X	X	X		
[49]	X		X		
[89]		X		X	
[99]	X	X	X		
[100]		X			
[50]		X			
[101]	X	X	X	X	
[102]		X			
[103]		X			
[104]		X	X	X	
[105]	X		X		
[106]		X	X		X
[88]			X	X	
[51]	X		X		
[107]		X			
[108]			X		
[109]			X		
[110]	X		X		X
[111]	X	X	X	X	
[112]	X	X	X		
[90]		X	X		
[113]	X	X	X		
[114]	X				
[115]	X			X	

**Table 14:** User interaction modalities.

Reference	Gesture	Speech	Gaze	GUI	TUI
[92]				X	
[91]				X	
[93]	X				
[94]	X				
[48]		X			
[95]	X				
[96]				X	
[97]				X	
[98]		X		X	
[49]	X				
[89]				X	X
[99]		X		X	
[100]	X	X			
[50]	X	X			
[101]	X	X			
[102]	X			X	
[103]	X	X	X		
[104]	X				
[105]		X		X	
[106]					
[88]	X	X	X		
[51]	X				
[107]	X				
[108]					
[109]					
[110]	X				
[111]	X				
[112]	X				
[90]	X				
[113]	X	X			
[114]	X	X		X	
[115]	X				

**Table 15:** Evaluation methods.

Reference	Quantitative	Qualitative
[93]	Task completion time Number of help interventions Number of wrong actions performed	After-Scenario Questionnaire
[48]		SUS Questionnaire
[49]	Task completion time Percentage of correctly performed subtasks	Customized Questionnaire for subjective measurement Additional comments
[89]	Task completion time Number of help interventions	SUS Questionnaire NASA-TLX Questionnaire Customized usability questionnaire
[99]	Production cycle time MURI for ergonomics	
[100]		Customized questionnaire for subjective measurement
[50]	Average applied force Applied force standard deviation	Customized usability Questionnaire Customized user experience Questionnaire
[103]	Stress level Concentration level Task/attention switching	SART test NASA-RTLX
[105]	Production cycle time operators saturation level	
[106]		Verbal feedback
[88]	Task execution time Amount of robot usage	NASA-TLX Questionnaire Additional comments
[51]	Task completion time Robot idle time	Customized questionnaires for subjective measurement Additional comments
[107]	RULA for ergonomics	NASA-TLX Questionnaire
[108]	Proximity to the robot Galvanic Skin Response (GSR)	Customized trust assessment questionnaire
[90]	Task completion time Robot idle time	SUS questionnaire Customized questionnaire for subjective measurement
[113]		SUS questionnaire Additional comments
[115]	RULA for ergonomics Task completion time	Satisfaction questionnaires

## Data Availability

All data are contained within the manuscript. Raw data are available from the corresponding author upon request.

## Abbreviations

The following abbreviations are used in this manuscript:

ACM	Association for Computing Machinery
ACM/IEEE—HRI	Association for Computing Machinery/Institute of Electrical and Electronics Engineers International Conference on Human–Robot Interaction
API	Application Programming Interface
AR	Augmented Reality
ASQ	After-Scenario Questionnaire
CIE	International Conference on Computers & Industrial Engineering
CIRP Ann.—Manuf. Technol.	College International pour la Recherche en Productique Annals Manufacturing Technology
*CS*	CiteScore
Ebsco	Elton B. Stephens Company
GSR	Galvanic Skin Response
GUI	Graphical User Interface
HHD	Hand-Held Displays
HMC	Human–Machine Collaboration
HMD	Head-Mounted Displays
HRC	Human–Robot Collaboration
HRI	Human–Robot Interaction
IEEE ETFA	Institute of Electrical and Electronics Engineers International Conference on Emerging technologies and Factory Automation
IEEE ICAICA	Institute of Electrical and Electronics Engineers International Conference on Artificial Intelligence and Computer Applications
IEEE IROS	Institute of Electrical and Electronics Engineers International Conference on Robot & Human Interactive Communication
IEEE IWOBI	Institute of Electrical and Electronics Engineers International Work Conference on Bioinspired Intelligence
IEEE RO-MAN	Institute of Electrical and Electronics Engineers International Conference on Robot & Human Interactive Communication
IEEE Robot. Autom. Lett.	Institute of Electrical and Electronics Engineers Robotics and Automation Letters
IEEE Robot. Autom. Mag.	Institute of Electrical and Electronics Engineers Robotics & Automation Magazine
IFIP—AICT	International Federation for Information Processing - Advances in Information and Communication Technology
Int. J. Adv. Manuf. Syst.	International Journal of Advanced Manufacturing Technology
ISARC	International Symposium on Automation and Robotics in Construction
ISO	International Organization for Standardization
JCR	Journal Citation Reports
LCD	Liquid Crystal Display
LED	Light-Emitting Diode
LNCS	Lecture Notes in Computer Science
MR	Mixed Reality
NASA-TLX	National Aeronautics and Space Administration Task Load Index
NASA-RTLX	National Aeronautics and Space Administration Raw Task Load Index
PICOC	Population, Intervention, Comparison, Outcome, and Context
QC	Quality Criteria
Robot. Comput. Integr. Manuf.	Robotics and Computer-Integrated Manufacturing
RULA	Rapid Upper Limb Assessment
SAE	Society of Automotive Engineers
SART	Sustained Attention to Response TASK
SJR	Scimago Journal Rank
SLR	Systematic Literature Review
SUS	System Usability Scale
TUI	Tangible User Interface
UHP	Ultra High Performance lamp
UHP—DLP	Ultra High Performance lamp—Digital light processing
UR	Universal Robots
VR	Virtual Reality
XR	Extended Reality
WoS	Web of Science
